# Do iron homeostasis biomarkers mediate the associations of liability to type 2 diabetes and glycemic traits in liver steatosis and cirrhosis: a two-step Mendelian randomization study

**DOI:** 10.1186/s12916-024-03486-w

**Published:** 2024-06-26

**Authors:** Ying Liang, Shan Luo, Steven Bell, Jacky Man Yuen Mo, Baoting He, Yangzhong Zhou, Xiaoyin Bai, Shiu Lun Au Yeung

**Affiliations:** 1 School of Public Health, LKS Faculty of Medicine, The University of Hong Kong, Hong Kong SAR, China; 2 Precision Breast Cancer Institute, Department of Oncology, University of Cambridge, Cambridge, UK; 3 Cancer Research UK Cambridge Institute, Li Ka Shing Centre, University of Cambridge, Cambridge, UK; 4 Department of Rheumatology, Peking Union Medical College Hospital, National Clinical Research Center for Dermatologic and Immunologic Diseases (NCRC-DID), Beijing, 100730 China; 5 Department of Gastroenterology, Peking Union Medical College Hospital, Peking Union Medical College and Chinese Academy of Medical Sciences, Beijing, 100730 China

**Keywords:** Type 2 diabetes, Glycemic traits, Insulin, Ferritin, Liver iron, Liver steatosis, Liver cirrhosis

## Abstract

**Background:**

Previous studies, including Mendelian randomization (MR), have demonstrated type 2 diabetes (T2D) and glycemic traits are associated with increased risk of metabolic dysfunction-associated steatotic liver disease (MASLD). However, few studies have explored the underlying pathway, such as the role of iron homeostasis.

**Methods:**

We used a two-step MR approach to investigate the associations of genetic liability to T2D, glycemic traits, iron biomarkers, and liver diseases. We analyzed summary statistics from various genome-wide association studies of T2D (*n* = 933,970), glycemic traits (*n* ≤ 209,605), iron biomarkers (*n* ≤ 246,139), MASLD (*n* ≤ 972,707), and related biomarkers (alanine aminotransferase (ALT) and proton density fat fraction (PDFF)). Our primary analysis was based on inverse-variance weighting, followed by several sensitivity analyses. We also conducted mediation analyses and explored the role of liver iron in post hoc analysis.

**Results:**

Genetic liability to T2D and elevated fasting insulin (FI) likely increased risk of liver steatosis (OR_liability to T2D_: 1.14 per doubling in the prevalence, 95% CI: 1.10, 1.19; OR_FI_: 3.31 per log pmol/l, 95% CI: 1.92, 5.72) and related biomarkers. Liability to T2D also likely increased the risk of developing liver cirrhosis. Genetically elevated ferritin, serum iron, and liver iron were associated with higher risk of liver steatosis (OR_ferritin_: 1.25 per SD, 95% CI 1.07, 1.46; OR_liver iron_: 1.15 per SD, 95% CI: 1.05, 1.26) and liver cirrhosis (OR_serum iron_: 1.31, 95% CI: 1.06, 1.63; OR_liver iron_: 1.34, 95% CI: 1.07, 1.68). Ferritin partially mediated the association between FI and liver steatosis (proportion mediated: 7%, 95% CI: 2–12%).

**Conclusions:**

Our study provides credible evidence on the causal role of T2D and elevated insulin in liver steatosis and cirrhosis risk and indicates ferritin may play a mediating role in this association.

**Supplementary Information:**

The online version contains supplementary material available at 10.1186/s12916-024-03486-w.

## Highlights

• Ferritin possibly mediates the association of insulin resistance in liver steatosis. Targeting the reduction of ferritin may mitigate the risk of liver steatosis arising from elevated insulin.

• Liver iron is likely to be positively associated with liver steatosis and the irreversible stage, liver cirrhosis, and mediates the association of ferritin and liver steatosis.

• Genetically elevated HbA1c is shown to decrease the risk of liver steatosis due to its erythrocytic property. Caution should be applied when using HbA1c values for MASLD management.

**Supplementary Information:**

The online version contains supplementary material available at 10.1186/s12916-024-03486-w.

## Background

Metabolic dysfunction-associated steatotic liver disease (MASLD), formerly known as non-alcoholic fatty liver disease [1], is a significant worldwide health issue that affects approximately 32% of the population [2]. Despite its widespread prevalence, effective treatments for MASLD remain elusive [2]. The spectrum of MASLD ranges from steatosis with > 5% hepatic fat accumulation, and progression to steatohepatitis with ballooning, inflammation, fibrosis, and even liver cirrhosis [3]. The presence of MASLD is related to other major chronic diseases, notably type 2 diabetes (T2D) and insulin resistance, although evidence from earlier studies suggests that this association may be bi-directional [4, 5]. Among possible mechanisms linking these two diseases, ferritin (a protein in cells for iron storage which can be found in hepatocytes) has been proposed to play a role in hepatic steatosis, inflammation, and fibrosis [6]. Meta-analyses of observational studies have shown people with T2D have higher ferritin, which is a risk factor for the severity of advanced fibrosis [7, 8]. Despite these postulated pathways, few studies have investigated the mediating role of ferritin and other iron homeostasis biomarkers in the associations of T2D or glycemic traits in MASLD. However, these studies were mainly observational, and hence are vulnerable to confounding and reverse causation [9, 10].

Mendelian randomization (MR), a study design that relies on genetic variants randomly allocated at conception, is increasingly used to investigate etiologic questions given that it is less prone to confounding than conventional observational studies [11], and tends to give more consistent results with randomized controlled trials, such as the lack of association of HDL-cholesterol in coronary artery disease [12]. Previous MR studies have revealed that genetically elevated fasting insulin (FI) and liability to T2D increase alanine aminotransferase (ALT) and MASLD risk [13–15], whereas some MR studies have shown higher systemic iron status and iron homeostasis biomarkers (e.g., ferritin, serum iron, or transferrin saturation (TSAT)) may increase risk of MASLD [16, 17], but with some exceptions [18, 19]. These discrepancies could be driven by a lack of statistical power, or possible biases including highly pleiotropic variants (e.g., rs1800562 in *HFE*) which relate to the predominant type of hemochromatosis [20]. A recent MR study found genetic liability to T2D and glycemic traits may impact iron homeostasis biomarkers, particularly ferritin, whilst the association of most iron homeostasis biomarkers with T2D was unlikely causal [21]. This suggests a possible mechanistic pathway in which hyperglycemia and elevated insulin relate to MASLD. Although, to the best of our knowledge, this has not been explored using an MR design. Therefore, we conducted a two-step MR study to comprehensively assess the mediating role of different iron homeostasis biomarkers in the associations of genetic liability to T2D and glycemic traits in liver steatosis, liver cirrhosis and its biomarkers (ALT and magnetic resonance imaging (MRI)-derived proton density fat fraction (PDFF)) [22, 23]. We additionally included MRI-derived liver iron as a possible mediator to explore the potential differential effects compared to blood-based measures of iron homeostasis.

## Methods

### Study design

**Fig. 1:**
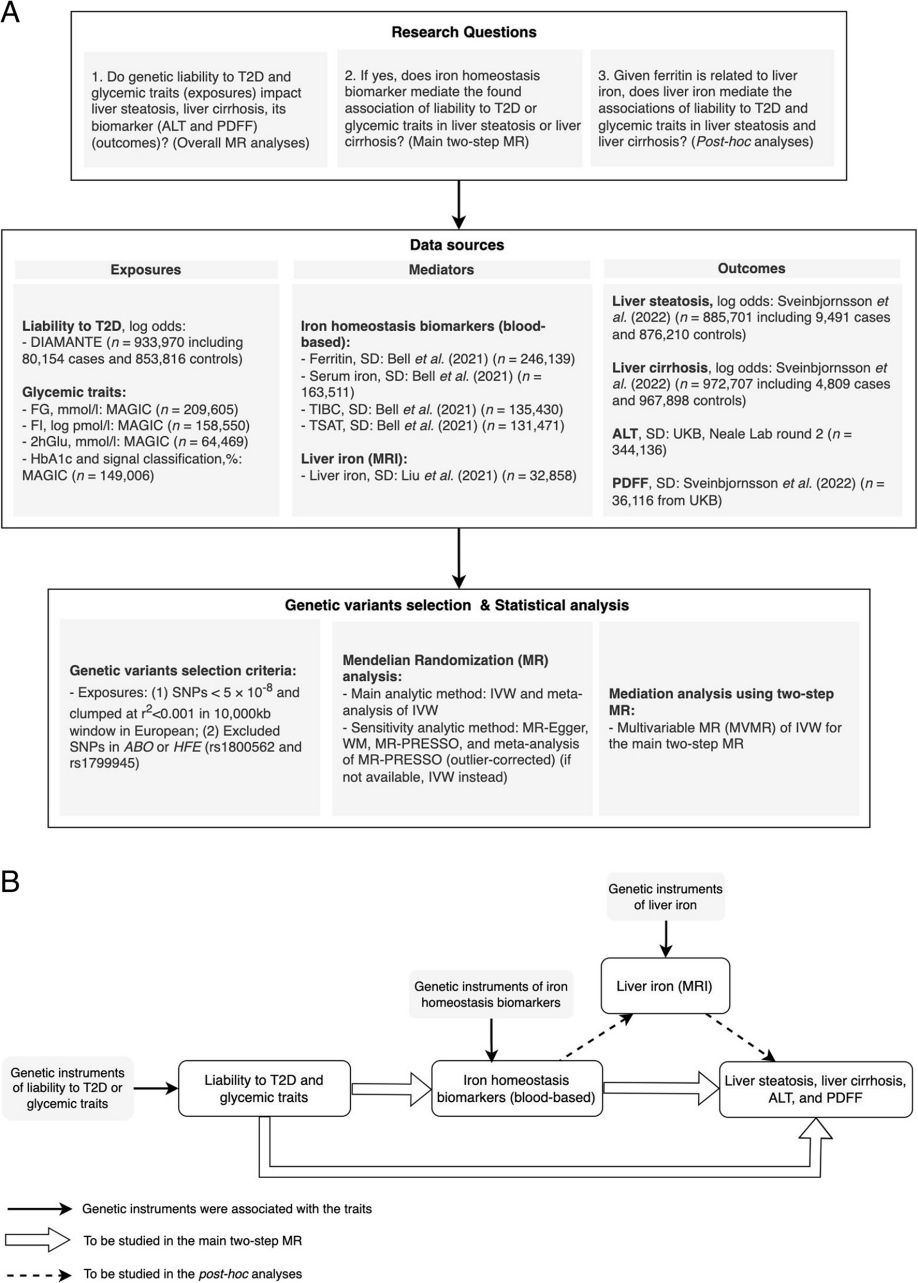
Study design of this study and diagram of this two-step Mendelian randomization analysis. **A** Study design of this study. **B** Diagram of this two-step Mendelian randomization. Liability to T2D, liability to type 2 diabetes; FG, fasting glucose; FI, fasting insulin; 2hGlu, 2-hour glucose; HbA1c, hemoglobin A1c; TIBC, total iron-binding capacity; TSAT, transferrin saturation; PDFF, proton density fat fraction; ALT, alanine aminotransferase. IVW, inverse-variance weighting; WM, weighted median; MR-PRESSO, Mendelian Randomization Pleiotropy RESidual Sum and Outlier

We adopted a two-step MR design using summary statistics from relevant genome-wide association studies (GWAS) (Fig. 1A). To estimate the overall effect, we first used a standard MR approach to evaluate the associations of liability to T2D and glycemic traits (exposures) on liver steatosis, liver cirrhosis and its biomarkers (outcomes) (Fig. 1B). We then extracted the associations of liability to T2D and glycemic traits (exposures) on iron homeostasis biomarkers (mediators) based on our previous study (Step 1, see Additional file 1: Table S1) [21]. Afterwards, we assessed the associations between these selected mediators and the liver-related outcomes (Step 2) (Fig. 1B). We also investigated the role of liver iron content in the associations of iron homeostasis biomarkers with the liver-related outcomes as a post hoc analysis (Fig. 1B). As with all MR analyses, there are three core assumptions, including relevance, independence, and exclusion restriction [23].

This study was conducted in accordance with the Strengthening the Reporting of Observational Studies in Epidemiology using MR (STROBE-MR) (Additional file 2: Supplementary Note) [24].

### Data sources

#### Genetic instruments for T2D, glycemic traits, iron homeostasis biomarkers, and liver iron as exposures

We selected genetic instruments for our study based on specific criteria. These instruments included single nucleotide polymorphisms (SNPs) that demonstrated strong associations with our exposure of interest, with a significance threshold of *P* < 5 × 10^−8^. We ensured that these SNPs were independent of each other, with a linkage disequilibrium (LD) threshold of *r*^2^ < 0.001. To reduce potential bias from population stratification, we exclusively used data from individuals of European ancestries. We gathered the genetic instruments for our study from several datasets. For T2D, we obtained data from the Diabetes Meta-Analysis of Trans-Ethics Associations Studies (DIAMANTE) Consortium, which encompassed 80,154 cases and 853,816 controls [25]. Additionally, we obtained information on glycemic traits, including fasting glucose (FG) measured in mmol/l (*n* = 209,605), 2-hour glucose (2hGlu) measured in mmol/l (*n* = 64,469), FI in log pmol/l (*n* = 158,550), and hemoglobin A1c (HbA1c) in percentage (*n* = 149,006) from the Meta-Analyses of Glucose and Insulin-related traits Consortium (MAGIC) [26]. Furthermore, we incorporated data on four iron homeostasis biomarkers, including ferritin (standard deviation (SD), *n* = 246,139), serum iron (SD, *n* = 163,511), total iron-binding capacity (TIBC) (SD, *n* = 135,430), and TSAT (SD, *n* = 131,471) from a recent GWAS meta-analysis [27], since these biomarkers represent different aspects of iron homeostasis including iron stores (serum ferritin) and iron transport (serum iron, TIBC, TSAT) (Additional file 1: Tables S2). As part of post hoc analysis, we also included MRI-derived liver iron content data from the UK Biobank (UKB; SD, *n* = 32,858) [28]. To mitigate potential bias stemming from horizontal pleiotropy and distinguish the effects of iron in general from hereditary hemochromatosis, we implemented a strategy to exclude genetic instruments in *ABO* and associated with *HFE*-hemochromatosis (specifically rs1800562 and rs1799945, if any) [20, 29]. Details of data sources and genetic instruments were listed in Additional file 1: Tables S3–S8.

#### Genetic associations for liver steatosis, liver cirrhosis, and related biomarkers as outcomes

Genetic associations of liver diseases were obtained from relevant GWAS involving individuals of European descent [30]. These studies included liver steatosis, which involved 9491 cases and 876,210 controls, and liver cirrhosis, which encompassed 4809 cases and 967,898 controls [30]. Biomarkers of liver diseases included ALT (SD, *n* = 344,136) [31], and PDFF (SD,* n* = 36,116) from UKB (Additional file 1: Table S9) [30]. For outcomes where the standard errors were not provided by authors (i.e., liver steatosis, liver cirrhosis, and PDFF), we calculated these using the corresponding betas and *P* values based on normal distribution.

Details of these GWAS, including the mean age, sex distribution, and covariates adjustment, are described in Additional file 1: Table S3 and Additional file 2: Supplementary Note. Information pertaining to potential participant overlap across the GWAS is listed in Additional file 1: Table S4. These data were harmonized based on the reported effect allele and effect allele frequencies (EAF). Palindromic variants with intermediate EAF (42% < EAF < 58%) were discarded to avoid ambiguity in the strand direction.

### Statistical analyses

#### Main analyses

We calculated the variance (*R*^2^) of the exposures explained by genetic instruments and approximated the overall F-statistic. An F-statistic > 10 suggests that weak instrument bias is unlikely [32]. We used inverse-variance weighting (IVW) with multiplicative random effects as the main analysis, which assumes balanced pleiotropy. We evaluated heterogeneity of the variant-specific Wald ratio through *I*^2^ where a high value may imply the presence of invalid instruments [33]. We performed random-effects meta-analysis for liver steatosis and liver cirrhosis as the corresponding GWAS did not provide pooled estimates across the studies for these outcomes [30]. Considering the interpretation of associations with liability to T2D (per log odds), we used a unit of per doubling in the prevalence instead by multiplying the estimates by 0.693 to improve the interpretation [34].

#### Sensitivity analyses

Various sensitivity analyses based on different assumptions were used to assess the robustness of the main findings. These included MR-Egger (assuming instrument strength independent of direct effect, InSIDE), weighted median (WM, assuming that the majority of selected instruments are valid), and Mendelian Randomization Pleiotropy RESidual Sum and Outlier (MR-PRESSO, outlier-robust) [35]. We also performed random-effects meta-analysis of MR-PRESSO (if available) for liver steatosis and liver cirrhosis as sensitivity analyses. The *P* value of MR-Egger intercept was used to assess overall horizontal pleiotropy. *I*^2^_GX_ statistic was used to detect the possibility of dilution bias of MR-Egger estimates [36]. Lastly, given that HbA1c is influenced by both glycemic and erythrocytic properties, we repeated the analyses regarding HbA1c by classifying the instruments according to the reported clusters (glycemia: 16 SNPs; erythrocytic: 44 SNPs (iron: 3 SNPs; mature red blood cell: 23 SNPs; reticulocyte: 18 SNPs); and unknown: 13 SNPs) based on the original GWAS (see footnote of Additional file 1: Table S10 for details on the classification) [26].

#### Mediation analyses

Multivariable MR (MVMR) analyses were also used to assess the observed association of mediators on outcomes, adjusting for exposures to control for horizontal pleiotropy [37]. The same method was also used for mediation analysis to assess the associations of exposures on outcomes adjusting for mediators to decompose the total effect into direct and indirect effects. The mediation effects were calculated by the product of coefficients methods, with the standard error derived using the delta method [38, 39].

### Power calculation

Based on the significance level of 0.05 and *R*^2^ of T2D, glycemic traits, iron homeostasis biomarkers, and liver iron (exposures) explained by the genetic instruments in the analyses, we estimated the effect sizes and odds ratios which we could detect at least 80% study power for the associations with iron homeostasis biomarkers, liver diseases, and related biomarkers (https://sb452.shinyapps.io/power/) [40], and are listed in Additional file 1: Table S11.

Details of these methods can be found in Additional file 2: Supplementary Note.

All analyses were performed using R version 4.2.2 with R packages (“*TwoSampleMR*” version 0.5.6 [41], “*MRPRESSO*” version 1.0, “*forestplot*” version 3.1.1, and “*msm*” version 1.7).

## Results

### Genetic instruments of liability to T2D, glycemic traits, iron homeostasis biomarkers, and liver iron 

In this study, we included up to 180 SNPs for liability to T2D (F-statistic: 246, *R*^2^: 4.5%), 66 SNPs for FG (F-statistic: 154, *R*^2^: 4.6%), 37 SNPs for FI (F-statistic: 58, *R*^2^: 1.3%), 13 SNPs for 2hGlu (F-statistic: 63, *R*^2^: 1.3%), 72 SNPs for HbA1c (F-statistic: 118, *R*^2^: 5.4%), 59 SNPs for ferritin (F-statistic: 77, *R*^2^: 1.8%), 27 SNPs for serum iron (F-statistic: 161, *R*^2^: 2.6%), 31 SNPs for TIBC (F-statistic: 162, *R*^2^:3.6%), 26 SNPs for TSAT (F-statistic: 188, *R*^2^: 3.6%), and six SNPs for liver iron (F-statistic: 299, *R*^2^: 5.2%). The F-statistics indicated low evidence of weak instrument bias (Additional file 1: Table S5).

### Associations of liability to T2D and glycemic traits in liver steatosis, liver cirrhosis, ALT, and PDFF

**Fig. 2:**
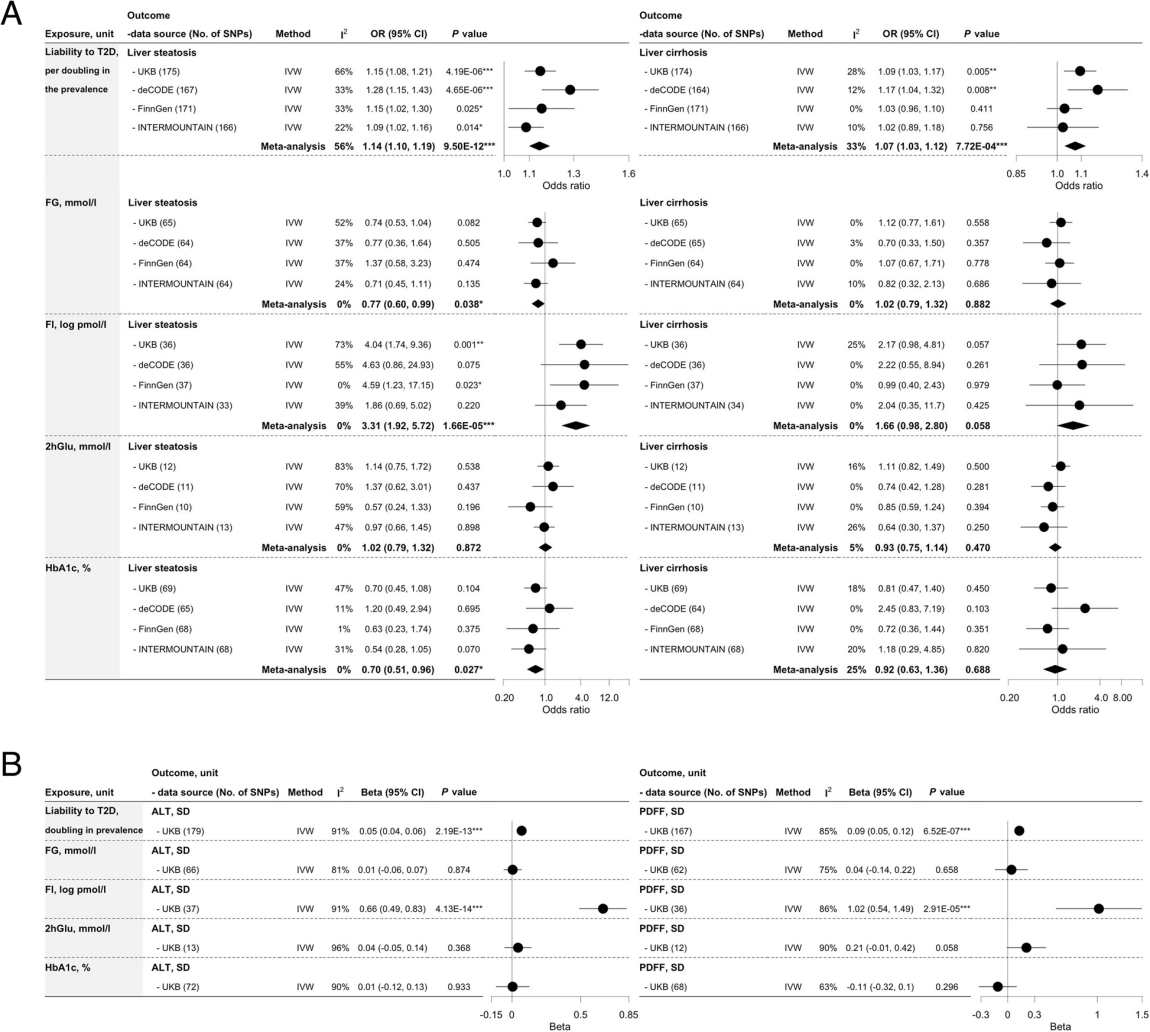
The associations of liability to type 2 diabetes and glycemic traits in liver steatosis, liver cirrhosis, alanine aminotransferase, and proton density fat fraction (MRI) using meta-analysis of inverse variance weighting. **A** The associations of liability of type 2 diabetes and glycemic traits in liver steatosis and liver cirrhosis. **B** The associations of type 2 diabetes and glycemic traits in alanine aminotransferase, and proton density fat fraction. Liver steatosis, log odds (*n* = 885,701 including 9491 cases and 876,210 controls) included UKB (5921 cases), deCODE (785 cases), FinnGen (651 cases), and INTERMOUNTAIN (2134 cases). Liver cirrhosis, log odds (*n* = 972,707 including 4809 cases and 967,898 controls) included UKB (2301 cases), deCODE (691 cases), FinnGen (1425 cases), and INTERMOUNTAIN (392 cases). Liability to T2D, liability to type 2 diabetes; FG, fasting glucose; FI, fasting insulin; 2hGlu, 2-hour glucose; HbA1c, hemoglobin A1c; ALT, alanine aminotransferase; PDFF, proton density fat fraction; No. of SNPs, number of single nucleotide polymorphisms; IVW, inverse-variance weighting; *I*^2^, degree of heterogeneity; 95% CI, 95% confidence interval. *P value < 0.05, **P value < 0.01, ***P value < 0.001

Liability to T2D and impaired FI likely increased risk of liver steatosis (odds ratio (OR)_liability to T2D_: 1.14 per doubling in the prevalence of T2D, 95% confidence interval (95% CI): 1.10 to 1.19); OR_FI_: 3.31 per log pmol/l, 95% CI: 1.92 to 5.72) as well as ALT and PDFF (Fig. 2). Liability to T2D also increased liver cirrhosis (OR_liability to T2D_: 1.07 per doubling in the prevalence, 95% CI: 1.03 to 1.12) (Fig. 2). These results were consistent with meta-analyses using MR-PRESSO estimates which were corrected for potential outliers (Additional file 3: Fig. S1). Although there were signs of heterogeneity based on *I*^2^ (e.g., ALT and PDFF), horizontal pleiotropy was not evident in most analyses and findings were generally consistent across sensitivity analyses (Fig. 2; Additional file 1: Table S12). However, the inverse association of HbA1c in liver steatosis (OR: 0.70 per %, 95% CI: 0.51 to 0.96) is likely driven by erythrocytic variants, which was also suggested by the HbA1c signal classification analyses (Additional file 3: Fig. S2; Additional file 1: Table S13). Based on MR-PRESSO analyses, scatter plots, and the forest plot, the inverse association of FG with liver steatosis was likely driven by rs1260326 (*GCKR*) in UKB (Additional file 1: Table S6; Additional file 3: Fig. S1, S3).

### Association of iron homeostasis biomarkers in liver steatosis, liver cirrhosis, ALT, and PDFF

**Fig. 3:**
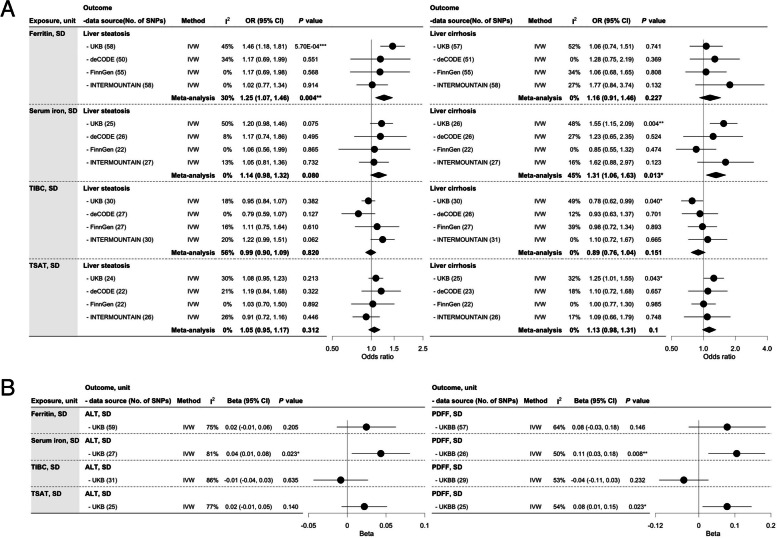
The associations of iron homeostasis biomarkers (*n* ≤ 246,139) in liver steatosis, liver cirrhosis, alanine aminotransferase, and proton density fat fraction using meta-analysis of inverse-variance weighting. **A** The associations of iron homeostasis biomarkers in liver steatosis and liver cirrhosis. **B** The associations of iron homeostasis biomarkers in alanine aminotransferase, and proton density fat fraction. Liver steatosis, log odds (*n* = 885,701 including 9491 cases and 876,210 controls) included UKB (5921 cases), deCODE (785 cases), FinnGen (651 cases), and INTERMOUNTAIN (2134 cases). Liver cirrhosis, log odds (*n* = 972,707 including 4809 cases and 967,898 controls) included UKB (2301 cases), deCODE (691 cases), FinnGen (1425 cases), and INTERMOUNTAIN (392 cases). TIBC, total iron-binding capacity; TSAT, transferrin saturation; ALT, alanine aminotransferase; PDFF, proton density fat fraction; No. of SNPs, number of single nucleotide polymorphisms; IVW, inverse-variance weighting; *I*^2^, degree of heterogeneity; 95% CI, 95% confidence interval. *P value < 0.05, **P value < 0.01, ***P value < 0.001

Ferritin was positively associated with liver steatosis (OR: 1.25 per SD, 95% CI: 1.07 to 1.46), with consistent findings from sensitivity analyses (Fig. 3; Additional file 3: Fig. S4; Additional file 1: Table S14). Serum iron was positively associated with liver cirrhosis (OR: 1.31 per SD, 95% CI: 1.06 to 1.63), ALT, and PDFF (Fig. 3). TSAT was positively associated with PDFF with directionally consistent estimates with other liver markers (Fig. 3; Additional file 1: Table S14). Heterogeneity was high across the analyses with variations in estimates across sensitivity analyses. Notably, the association concerning serum iron was attenuated in the MR-PRESSO analyses, likely driven by rs144861591 (*ZFP57*) (Additional file 3: Fig. S4; Additional file 1: Tables S7 and S14).

### Post hoc analyses concerning liver iron

**Fig. 4:**
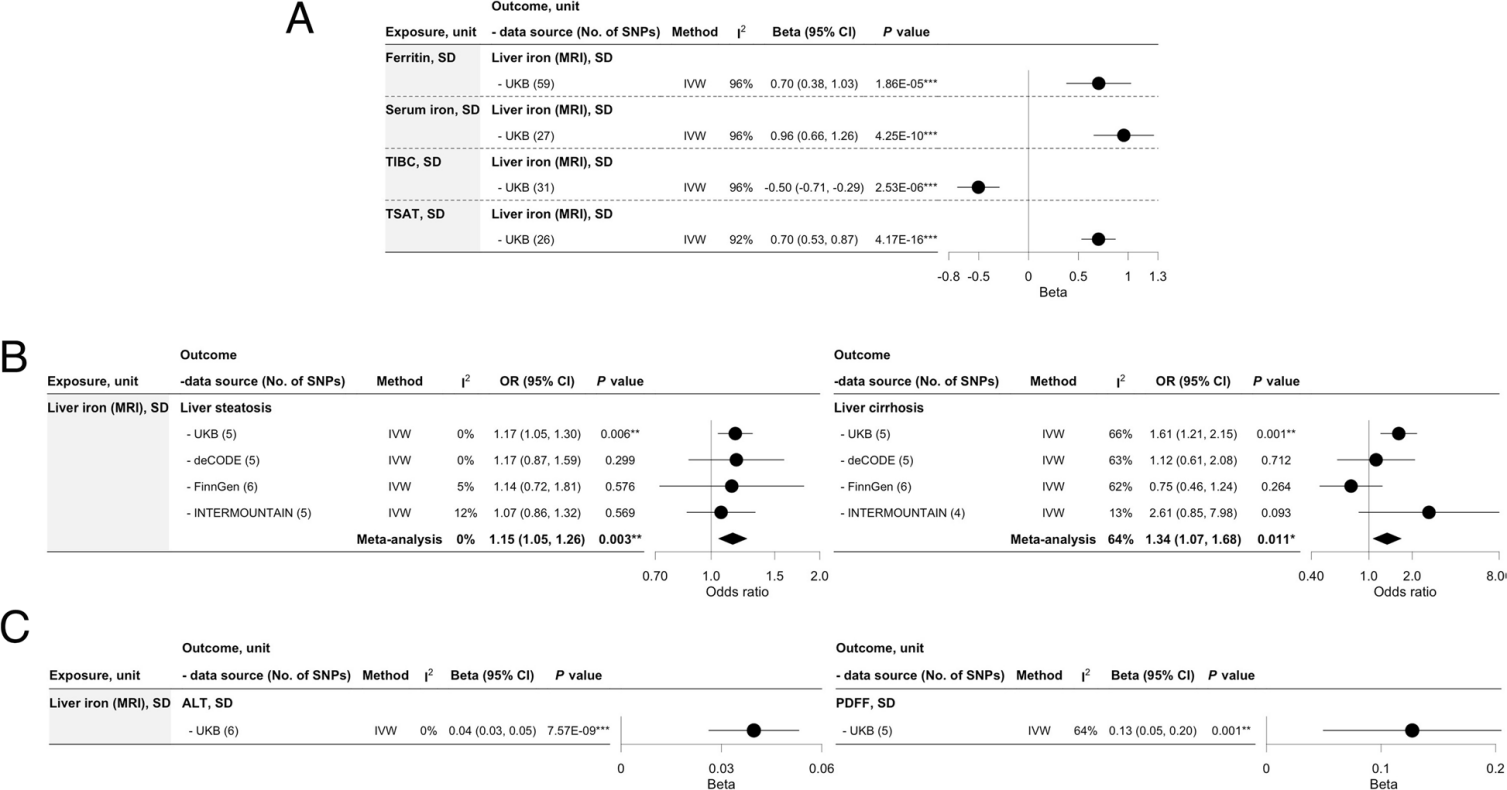
The associations of iron homeostasis biomarkers (blood-based) (*n* ≤ 246,139) in liver iron content (MRI) (*n* = 32,858, UKB) and the association of liver iron in liver steatosis, liver cirrhosis, alanine aminotransferase, and proton density fat fraction using Mendelian randomization. **A** The associations of iron homeostasis biomarkers in liver iron (MRI). **B** The associations of liver iron in liver steatosis and liver cirrhosis. **C** The associations of liver iron in alanine aminotransferase and proton density fat fraction alanine aminotransferase, and proton density fat fraction. Liver steatosis, log odds (*n* = 885,701 including 9491 cases and 876,210 controls) included UKB (5921 cases), deCODE (785 cases), FinnGen (651 cases), and INTERMOUNTAIN (2134 cases). Liver cirrhosis, log odds (*n* = 972,707 including 4809 cases and 967,898 controls) included UKB (2301 cases), deCODE (691 cases), FinnGen (1425 cases), and INTERMOUNTAIN (392 cases). TIBC, total iron-binding capacity; TSAT, transferrin saturation; ALT, alanine aminotransferase; PDFF, proton density fat fraction; No. of SNPs, number of single nucleotide polymorphisms; IVW, inverse-variance weighting; *I*^2^, degree of heterogeneity; 95% CI, 95% confidence interval. *P value < 0.05, **P value < 0.01, ***P value < 0.001

**Fig. 5:**
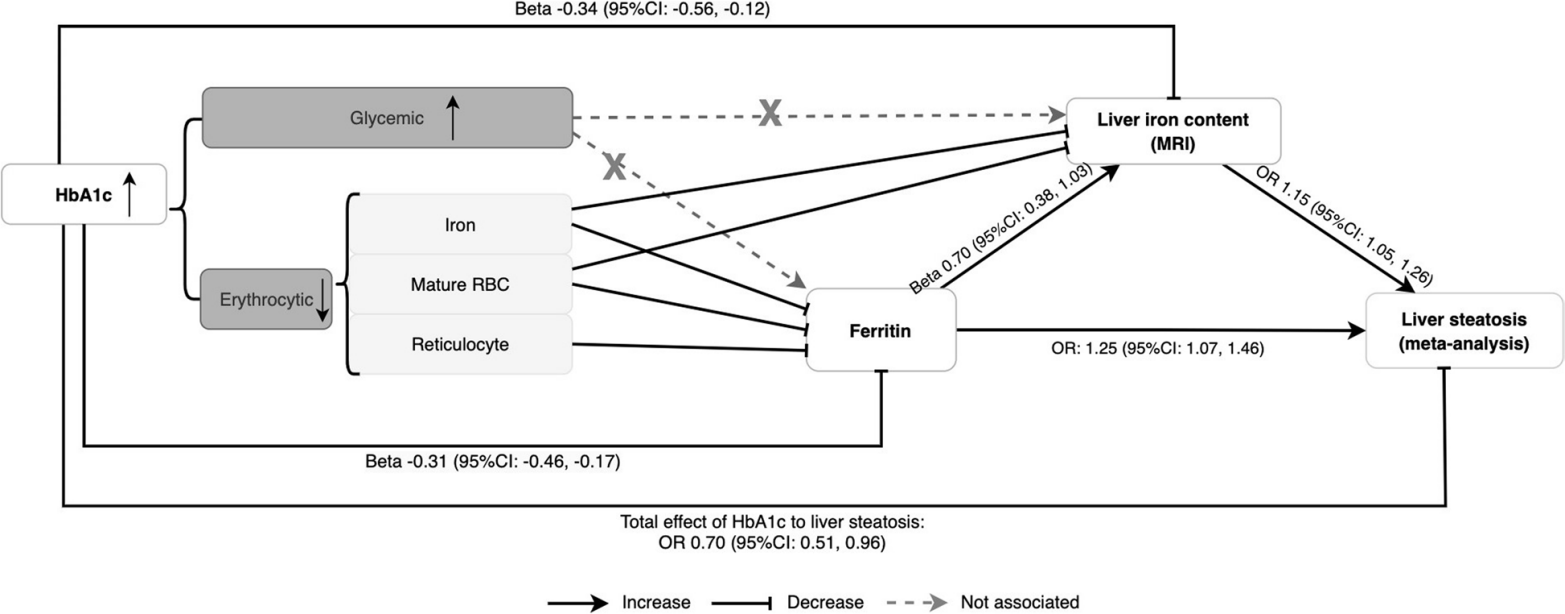
Summary of associations of HbA1c and its signal classification in ferritin, liver iron content (MRI), and liver steatosis (meta-analysis). HbA1c, hemoglobin A1c; Glycemic, probability of the variant in glycemic class, included fasting insulin, 2-hour glucose, and fasting glucose; Reticulocyte, probability of the variant in reticulocyte class, included reticulocyte count, reticulocyte fraction of red cells, immature fraction of reticulocytes, high light scatter reticulocyte count, and high light scatter reticulocyte percentage of red cells; Mature RBC, probability of the variant in mature red blood cell class, included red blood cell count, mean corpuscular volume, hematocrit, mean corpuscular hemoglobin, mean corpuscular hemoglobin concentration, hemoglobin concentration, and red cell distribution width; Iron, probability of the variant in iron class, included ferritin, transferrin, serum iron, and transferrin saturation; OR, odds ratio; 95% CI, 95% confidence interval

Ferritin, serum iron, and TSAT were positively associated with liver iron, and TIBC negatively with liver iron (Fig. 4A). Increased liver iron was associated with higher risk of liver steatosis (OR: 1.15 per SD, 95% CI: 1.05 to 1.26), liver cirrhosis (OR: 1.34 per SD, 95% CI: 1.07 to 1.68), ALT, and PDFF (Fig. 4B–C; Additional file 1: Tables S15–S16). However, liability to T2D or glycemic traits was not associated with liver iron, except for the inverse association of HbA1c in liver iron (*β*: -0.34 per percentage, 95% CI: − 0.56 to − 0.12) (Additional file 3: Fig. S5). However, the inverse association of HbA1c and liver steatosis via ferritin and liver iron was likely driven by erythrocytic property (Fig. 5; Additional file 3: Fig. S2; Additional file 1: Tables S13, S17).

### Findings from the mediation analyses

**Fig. 6:**
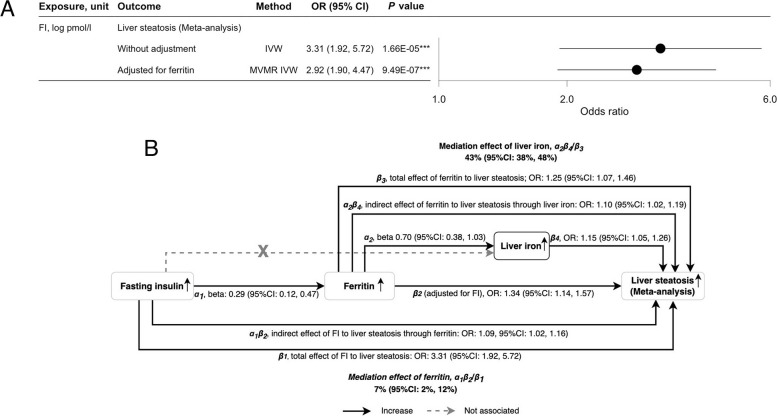
The associations of fasting insulin in liver steatosis with and without adjustment for ferritin, and summary of the mediating roles of ferritin and liver iron content in the associations of fasting insulin and liver steatosis (meta-analysis). **A** The associations of fasting insulin in liver steatosis with and without adjustment for ferritin using univariable and multivariable Mendelian randomization analysis. **B** Summary of the direct effects, indirect effects, and mediation effects of ferritin in the association of fasting insulin and steatosis, and of liver iron in the association of ferritin and steatosis (meta-analysis). FI, fasting insulin; HbA1c, hemoglobin A1c; MVMR, multivariable Mendelian randomization; IVW, inverse-variance weighting; OR, odds ratio; 95% CI, 95% confidence interval. *P value < 0.05, **P value < 0.01, ***P value < 0.001

Given that our previous study indicated FI was associated with ferritin (Step 1, Additional file 1: Table S1), and FI and ferritin were associated with liver steatosis (Figs. 2A and 3B), we subsequently assessed mediation using a two-step MR design. The corresponding mediation analysis showed ferritin partially mediated the association of FI in liver steatosis (Fig. 6A; Additional file 3: Fig. S6), where the proportion mediated via ferritin was 7% (95% CI: 2% to 12%) (Fig. 6B). As post hoc analysis, we also assessed the potential mediating role of liver iron between ferritin and liver steatosis and found evidence of partial mediation (proportion mediated: 43%, 95% CI: 38% to 48%) (Fig. 6B).

## Discussion

This is the largest MR study to investigate the associations of liability to T2D and glycemic traits in liver steatosis and liver cirrhosis. Our study is supported by previous observational and MR studies [4, 13–15, 42, 43] that genetic liability to T2D and FI likely increases the risk of liver steatosis and related biomarkers (e.g., ALT and PDFF), while liability to T2D appears to also increase the risk of liver cirrhosis. Our study also adds by showing, for the first time, iron homeostasis biomarkers and liver-iron content could potentially mediate some of these associations, hence providing genetic evidence that targeting the reduction of ferritin may mitigate the impact of MASLD risk arising from elevated insulin. Based on this study, we also clarify the paradoxical inverse association of HbA1c in liver steatosis is likely driven by the erythrocytic property of HbA1c and informs caution should be applied when using HbA1c values for disease management.

Previous investigations, including MR studies, have demonstrated that T2D and FI are associated with a higher MASLD risk, as well as increased levels of hepatic fat and ALT [4, 13–15, 42, 43], suggesting that the associations are likely causal. Similar findings were observed when extended to liver cirrhosis, an advanced stage of steatosis. The underlying mechanisms may include increased insulin resistance, proxied by elevated FI, leading to elevated free fatty acids uptake by liver, and hence increase de novo lipogenesis, lipolysis of dysfunctional adipose tissue, and subsequent risk of hepatic steatosis [3]. These findings imply that modifying risk factors of T2D and insulin resistance might protect against liver steatosis and its progression to liver cirrhosis.

Among the four iron homeostasis biomarkers considered, ferritin was most likely relevant to the development of liver steatosis, whilst serum iron likely increased ALT, a surrogate marker of MASLD. These findings are supported by previous observational and MR studies [9, 16, 17, 44, 45]. Increased ferritin may induce the deposition of hepatic iron in liver macrophages and stellate cells which results in hepatocellular damage and steatosis [6]. Although previous studies speculated that ferritin-MASLD association could be confounded by inflammation, this is not supported by clinical trials and mouse models [46, 47], and hence unlikely explains our findings, especially since MR design is more resistant to confounding compared to conventional observational studies [48]. Elevated serum iron levels may also promote oxidative stress through the production of free radicals in the liver through presence of excess iron [44]. Also, iron overload and high-iron diet are related to ferroptosis, an iron-dependent form of cell death caused by lipid peroxidation, which is a phenomenon related to MASLD progression [49, 50]. Hence, focusing on anti-ferroptosis therapy may help reverse the detrimental effects of elevated insulin in MASLD mediated via ferroptosis, such as *Malic Enzyme 1* (*ME1*) [51], although this requires further investigations using population-based studies. Furthermore, our mediation analysis adds to this hypothesis by suggesting that ferritin partially mediates the association of FI in liver steatosis and hence could be a possible target of intervention amongst people with T2D. However, the positive associations of serum iron in liver cirrhosis and PDFF were attenuated after removing rs144861591 which was strongly related to hereditary hemochromatosis (rs1800562 in *HFE*, *r*^2^ = 0.98) [41]. Although the MR-Egger intercept analyses suggested weak evidence of bias from horizontal pleiotropy, we are uncertain whether these associations are entirely independent of hemochromatosis, which warrants further research. Furthermore, the estimates were directionally consistent across different cohorts, which required verification when larger MASLD GWAS become available.

From our post hoc analyses, iron homeostasis biomarkers associated with liver iron were associated with a higher risk of MASLD and its biomarkers, and liver iron partially mediated the association of ferritin and liver steatosis, which is consistent with previous observational and MR studies [19, 52, 53]. The strong correlation between iron homeostasis biomarkers and liver iron is somewhat expected given that the liver is the main organ for iron storage [54]. Although studies on liver iron and MASLD are generally fewer compared to those of iron homeostasis biomarkers, evidence exists to suggest liver iron content is a strong predictor of liver disease severity [55], with possible pathways including increased inflammation, oxidative stress, and lipid oxidation [56]. Although we did not observe any association of genetic liability to T2D or glycemic traits in liver iron (apart from HbA1c which is likely driven by iron and mature red blood cells, i.e., iron recycling from senescent red blood cells) [21], which is consistent with a recent longitudinal study [42]. However, additional studies should explore whether sex-specific effects exist, as suggested by a previous longitudinal investigation [42].

The inverse association of FG in liver steatosis was inconsistent with the findings of liability to T2D, insulin, and glycemic signal class of HbA1c. Although these glycemic traits are well-characterized risk factors for T2D, recent studies indicated that they may have distinct relations with metabolomic signatures and CVD risk [57, 58]. However, our sensitivity analysis suggested this paradoxical finding could be driven by rs1260326 (*GCKR*), which encodes glucokinase regulatory protein (GKRP) (a primarily liver-specific protein) and is linked to decreased blood glucose by altering GKRP’s function of suppressing glucokinase activity and enhancing hepatic glycolysis, and concurrently increase total hepatic triglycerides [59], resulting in the risk allele of rs1260326 (T) decreasing the risk of T2D but increasing liver steatosis [30], and impacting the overall analyses. Although any effects observed for this *GCKR* risk variant could be arguably the downstream effects of glucose level variation (i.e., vertical pleiotropy and hence valid), given its high relevance in glucose metabolism, the increased circulating lipids could also arguably as an effect independent of glucose (i.e., horizontal pleiotropy and hence invalid) [60, 61]. As the overall inverse association is also most apparent in UKB, this may also be a reflection of selection bias distorting genetic associations in this cohort [62]. Given these issues, the paradoxical findings of glucose with liver steatosis should be interpreted with caution. Similarly, the unexpected inverse association of HbA1c and liver steatosis, although observed in a previous study [63], may be a reflection of the complex properties of HbA1c. The level of HbA1c is impacted by hyperglycemia and iron deficiency, where an earlier study showed iron deficiency can shift the distribution of HbA1c [64]. This is supported by the lack of association of HbA1c with liver steatosis when we restricted our analyses to only glycemia-related instruments (Fig. 5) and is more consistent with the glucose analyses without *GCKR* variant. These findings illustrated the challenges of using HbA1c solely as a reflection of hyperglycemia and are largely relevant to diabetes management.

Despite using an MR study design which is less prone to confounding than conventional observational studies, limitations remain. First, MR has stringent, unverifiable assumptions. Whilst confounding is less likely given genetics are randomly allocated at conception and with the use of GWAS which controls for population stratification, the possibility of exclusion restriction violation cannot be ruled out completely. Nevertheless, we applied different statistical methods with various assumptions that yielded comparable results. We also did not include variants which could be highly pleiotropic, such as the variants harboring *HFE*-hemochromatosis (rs1800562/rs1799945) and *ABO* gene region as per our previous study [21]. Although there was participant overlap in some of the GWAS used, which may bias our estimates towards the observational association in instances where weak instruments are used, this is unlikely given the large F-statistics used. Second, although we mainly focused on the overall analyses which combined different cohorts to maximize statistical power, these individual cohorts did not always give consistent associations (e.g., the associations of FI in liver cirrhosis, Fig. 2A). These discrepancies may have been driven by multiple factors such as participant selection criteria, or statistical power, and variation in genetic architecture in the underlying populations [65]. Third, we did not explore potential sex differences given the lack of sex-specific GWAS summary statistics whereas early studies suggested possible sex-specific associations of glycemic traits in liver iron and liver fat [42]. Additional studies with individual-level data (e.g., UK Biobank) might be helpful to address this limitation. Fourth, the effect sizes of liver steatosis and cirrhosis may be underestimated as the case definition in the MASLD GWAS is only based on ICD-10 diagnostic codes and hence may have missed patients who were symptomless and hence not being diagnosed in clinical settings [30], and UKB participants are more healthy and hence are vulnerable to selection bias [66]. However, our findings were directionally consistent with previous MR studies of liability to T2D, glycemic traits, and iron biomarkers in MASLD using GWAS with liver biopsy specimens definition [14, 16, 17, 19, 67]. Lastly, we only included studies of European ancestry. Whether the observed associations extend to other ethnic groups warrants further investigation.

## Conclusions

Our study highlights the role of T2D and FI in liver steatosis and cirrhosis etiology, and provides mechanistic insights into the mediating role of ferritin in the positive association of FI with liver steatosis. Whether targeting the reduction of ferritin in those with increased insulin would reduce the risk of MASLD requires further bespoke investigation.

### Supplementary Information

Additional file 1:  Table S1. The associations of liability to type 2 diabetes and glycemic traits in iron homeostasis biomarkers in our previous study (Liang et al., 2023). Table S2. Description of iron homeostasis biomarkers. Table S3. Information of the data sources used in this study. Table S4. Participant overlap information in the genome-wide associations studies used in this study. Table S5. The genetic instruments information of type 2 diabetes, glycemic traits, iron homeostasis biomarkers, and liver iron after harmonization. Table S6. Genetic instruments information of liability to type 2 diabetes and glycemic traits as exposures. Table S7. Genetic instruments information of iron homeostasis biomarkers as exposures. Table S8. Genetic instruments information of liver iron as exposures. Table S9. Genetic instruments information of liver steatosis, liver cirrhosis, ALT, PDFF, and liver iron as outcomes. Table S10. Genetic information of HbA1c signal classification. Table S11. Study power of this two-step Mendelian randomization study. Table S12. The associations of liability to type 2 diabetes and glycemic traits in liver steatosis, liver cirrhosis, alanine aminotransferase, and proton density fat fraction using Mendelian randomization analysis. Table S13. The associations of HbA1c signal classification in liver steatosis, liver cirrhosis, alanine aminotransferase, and proton density fat fraction using Inverse-variance weighting. Table S14. The associations of iron homeostasis biomarkers in liver steatosis, liver cirrhosis, alanine aminotransferase, and proton density fat fraction using Mendelian randomization analysis. Table S15. The associations of iron homeostasis biomarkers in liver iron using Mendelian randomization analysis. Table S16. The associations of liver iron in liver steatosis, liver cirrhosis, alanine aminotransferase, and proton density fat fraction using Mendelian randomization analysis. Table S17. The associations of HbA1c signal classification in iron homeostasis biomarkers and liver iron using Inverse-variance weighting.

Additional file 2:  Supplementary Note (STROBE-MR checklist and supplementary information of data sources).

Additional file 3:  Fig. S1 The associations of liability to type 2 diabetes and glycemic traits in liver steatosis and liver cirrhosis using meta-analysis of Inverse-variance weighting and MR-PRESSO (outlier-corrected). Fig. S2 The associations of HbA1c signal classifications in liver steatosis, ferritin, and liver iron using Mendelian randomization. Fig. S3 (a) Scatter plots and (b) forest plot of fasting glucose and liver steatosis in UK Biobank, deCODE, FinnGen, and INTERMOUNTAIN. Fig. S4 The associations of iron homeostasis biomarkers in liver steatosis and liver cirrhosis using meta-analysis of Inverse-variance weighting and MR-PRESSO (outlier-corrected). Fig. S5 The association of liability to type 2 diabetes and glycemic traits in liver iron (MRI) using Inverse-variance weighting. Fig. S6 The associations of fasting insulin, ferritin, and liver iron in liver steatosis with mediators and exposures adjustment using multivariable Mendelian randomization analysis.

## Data Availability

All data used in this study can be found in the cited references and the URLs in the Acknowledgements and Supplementary Materials.

## Abbreviations

2hGlu: 2-hour glucose
ALT: Alanine aminotransferase
DIAMANTE: Diabetes Meta-Analysis of Trans-Ethics associations studies
EAF: Effect allele frequencies
FG: Fasting glucose
FI: Fasting insulin
GWAS: Genome-wide association studies
HbA1c: Hemoglobin A1c
InSIDE: Instrument Strength Independent of Direct Effect
IVW: Inverse-variance weighting
LD: Linkage disequilibrium
MAGIC: Meta-Analyses of Glucose and Insulin-related traits Consortium
MASLD: Metabolic dysfunction-associated steatotic liver disease
MR: Mendelian randomization
MR-PRESSO: Mendelian Randomization Pleiotropy RESidual Sum and Outlier
MVMR: Multivariable Mendelian randomization
PDFF: Proton density fat fraction
R^2^: Variance of the exposures explained by genetic instruments
SD: Standard deviation
SNPs: Single nucleotide polymorphisms
STROBE-MR: Strengthening the Reporting of Observational Studies in Epidemiology using Mendelian randomization
T2D: Type 2 diabetes
TIBC: Total iron-binding capacity
TSAT: Transferrin saturation
UKB: UK Biobank
WM: Weighted median
