# Supplementary material for: Do iron homeostasis biomarkers mediate the associations of liability to type 2 diabetes and glycemic traits in liver steatosis and cirrhosis: a two-step Mendelian randomization study
Source: BMC Med. 2024 Jun 26;22:270. doi: 10.1186/s12916-024-03486-w (PMC11210020; doi:10.1186/s12916-024-03486-w)
Supplement: Supplementary file 2 — Additional file 2: Supplementary Note (STROBE-MR checklist and supplementary information of data sources). [file 12916_2024_3486_MOESM2_ESM.pdf]

# Supplementary Note

## STROBE-MR checklist

| Item No. | Section | | Checklist item | Relevant text from manuscript |
|---|---|---|---|---|
| 1 | **TITLE and ABSTRACT** | | Indicate Mendelian randomization (MR) as the study's design in the title and/or the abstract if that is a main purpose of the study | Title and Abstract |
| | **INTRODUCTION** | | | |
| 2 | **Background** | | Explain the scientific background and rationale for the reported study. What is the exposure? Is a potential causal relationship between exposure and outcome plausible? Justify why MR is a helpful method to address the study question | Introduction |
| 3 | **Objectives** | | State specific objectives clearly, including pre-specified causal hypotheses (if any). State that MR is a method that, under specific assumptions, intends to estimate causal effects | Introduction paragraph #2 |
| | **METHODS** | | | |
| 4 | **Study design and data sources** | | Present key elements of the study design early in the article. Consider including a table listing sources of data for all phases of the study. For each data source contributing to the analysis, describe the following: | |
| | | a) | Setting: Describe the study design and the underlying population, if possible. Describe the setting, locations, and relevant dates, including periods of recruitment, exposure, follow-up, and data collection, when available. | Methods – Study design, data sources; Supplementary Note– Data sources; Table S3 |
| | | b) | Participants: Give the eligibility criteria, and the sources and methods of selection of participants. Report the sample size, and whether any power or sample size calculations were carried out prior to the main analysis | Methods – Study design, data sources; Supplementary Note– Data sources; Table S3; Figure 1A |
| | | c) | Describe measurement, quality control and selection of genetic variants | Data sources; Figure 1A |
| | | d) | For each exposure, outcome, and other relevant variables, describe methods of assessment and diagnostic criteria for diseases | Supplementary Note – Data sources |

| | | | | |
|---|---|---|---|---|
| | | e) | Provide details of ethics committee approval and participant informed consent, if relevant | Since this study relied on publicly available summary statistics from GWAS, ethics approval was not required. |
| 5 | **Assumptions** | | Explicitly state the three core IV assumptions for the main analysis (relevance, independence and exclusion restriction) as well assumptions for any additional or sensitivity analysis | Methods – Study design |
| 6 | **Statistical methods: main analysis** | | Describe statistical methods and statistics used | |
| | | a) | Describe how quantitative variables were handled in the analyses (i.e., scale, units, model) | Methods – Statistical analyses |
| | | b) | Describe how genetic variants were handled in the analyses and, if applicable, how their weights were selected | Methods – Statistical analyses (main analyses and sensitivity analyses) |
| | | c) | Describe the MR estimator (e.g. two-stage least squares, Wald ratio) and related statistics. Detail the included covariates and, in case of two-sample MR, whether the same covariate set was used for adjustment in the two samples | Methods – Statistical analyses (main analyses and sensitivity analyses) |
| | | d) | Explain how missing data were addressed | NA |
| | | e) | If applicable, indicate how multiple testing was addressed | Methods – Statistical analyses (Mediation analyses) |
| 7 | **Assessment of assumptions** | | Describe any methods or prior knowledge used to assess the assumptions or justify their validity | Introduction, Figure 1B |
| 8 | **Sensitivity analyses and additional analyses** | | Describe any sensitivity analyses or additional analyses performed (e.g. comparison of effect estimates from different approaches, independent replication, bias analytic techniques, validation of instruments, simulations) | Methods – Statistical analyses (sensitivity analyses); Figure 1A |
| 9 | **Software and pre-registration** | | | |
| | | a) | Name statistical software and package(s), including version and settings used | Methods (last paragraph) |
| | | b) | State whether the study protocol and details were pre-registered (as well as when and where) | NA |

| | | | | |
|---|---|---|---|---|
| | **RESULTS** | | | |
| 10 | **Descriptive data** | | | |
| | | a) | Report the numbers of individuals at each stage of included studies and reasons for exclusion. Consider use of a flow diagram | Supplementary Note 2 – Data source |
| | | b) | Report summary statistics for phenotypic exposure(s), outcome(s), and other relevant variables (e.g. means, SDs, proportions) | Results; Tables S5 – S9 |
| | | c) | If the data sources include meta-analyses of previous studies, provide the assessments of heterogeneity across these studies | Figures 2 – 4; Figures S1, S4, S6; Tables S11 – S16 |
| | | d) | For two-sample MR:<br>i. Provide justification of the similarity of the genetic variant-exposure associations between the exposure and outcome samples<br>ii. Provide information on the number of individuals who overlap between the exposure and outcome studies | i. $r^2$<0.001 within 10,000 kb n the 1000 genomes reference panel (European)<br>ii. Table S4 |
| 11 | **Main results** | | | |
| | | a) | Report the associations between genetic variant and exposure, and between genetic variant and outcome, preferably on an interpretable scale | Tables S6 – S10; |
| | | b) | Report MR estimates of the relationship between exposure and outcome, and the measures of uncertainty from the MR analysis, on an interpretable scale, such as odds ratio or relative risk per SD difference | Results; Figures 2 – 4; Figures S1-S2, S4-S6; Tables S11 – S16 |
| | | c) | If relevant, consider translating estimates of relative risk into absolute risk for a meaningful time period | NA |
| | | d) | Consider plots to visualize results (e.g. forest plot, scatterplot of associations between genetic variants and outcome versus between genetic variants and exposure) | Figures 2 – 4; Figures S1-S2, S4-S6 |
| 12 | **Assessment of assumptions** | | | |
| | | a) | Report the assessment of the validity of the assumptions | Results; Figures 2 – 4 ($I^2$); Figures S1, S4 – S6 ($I^2$); Tables S5 ($R^2$, F-statistic, $I^2_{GX}$), S11 – S16 ($I^2$, *P* value of MR-Egger intercept) |

| | | | | |
|---|---|---|---|---|
| | | b) | Report any additional statistics (e.g., assessments of heterogeneity across genetic variants, such as $I^2$, Q statistic or E-value) | Results; Figures 2 – 4 ($I^2$); Figures S1, S4 – S6 ($I^2$); Tables S11 – S16 ($I^2$, *P* value of MR-Egger intercept) |
| 13 | **Sensitivity analyses and additional analyses** | | | |
| | | a) | Report any sensitivity analyses to assess the robustness of the main results to violations of the assumptions | Tables S11, S13 -S15 (MR-Egger, weighted median; MR-PRESSO); Figures S1, S4 |
| | | b) | Report results from other sensitivity analyses or additional analyses | Results |
| | | c) | Report any assessment of direction of causal relationship (e.g., bidirectional MR) | Results – *Exploratory analyses* concerning liver iron; Figures 5 – 6; Figure S5 |
| | | d) | When relevant, report and compare with estimates from non-MR analyses | Discussion |
| | | e) | Consider additional plots to visualize results (e.g., leave-one-out analyses) | Scatter plots, Figure S3 |
| | **DISCUSSION** | | | |
| 14 | **Key results** | | Summarize key results with reference to study objectives | Discussion paragraph #1 |
| 15 | **Limitations** | | Discuss limitations of the study, taking into account the validity of the IV assumptions, other sources of potential bias, and imprecision. Discuss both direction and magnitude of any potential bias and any efforts to address them | Discussion paragraph #6 |
| 16 | **Interpretation** | | | |
| | | a) | Meaning: Give a cautious overall interpretation of results in the context of their limitations and in comparison with other studies | Discussion paragraph #2-5 |
| | | b) | Mechanism: Discuss underlying biological mechanisms that could drive a potential causal relationship between the investigated exposure and the outcome, and whether the gene-environment equivalence assumption is reasonable. Use causal language carefully, clarifying that IV estimates may provide causal effects only under certain assumptions | Discussion paragraph #2-5 |

| | | | |
|---|---|---|---|
| | | c) Clinical relevance: Discuss whether the results have clinical or public policy relevance, and to what extent they inform effect sizes of possible interventions | Conclusions |
| 17 | **Generalizability** | Discuss the generalizability of the study results (a) to other populations, (b) across other exposure periods/timings, and (c) across other levels of exposure | Discussion paragraph #6 |
| | **OTHER INFORMATION** | | |
| 18 | **Funding** | Describe sources of funding and the role of funders in the present study and, if applicable, sources of funding for the databases and original study or studies on which the present study is based | This study was partly funded by the Health and Medical Research Fund, Food and Health Bureau, HKSAR Government, Hong Kong, China (CFS-HKU1). The funder had no role in the design, analyses, interpretation of results or writing of the paper. |
| 19 | **Data and data sharing** | Provide the data used to perform all analyses or report where and how the data can be accessed, and reference these sources in the article. Provide the statistical code needed to reproduce the results in the article, or report whether the code is publicly accessible and if so, where | All data used in this study can be found in the cited references and the URLs in the Acknowledgements and Supplementary Materials. |
| 20 | **Conflicts of Interest** | All authors should declare all potential conflicts of interest | SLAY received honoraria from SomaLogic for scientific presentations on proteomic studies that was unrelated to this study. The authors declare that they have no other competing interests. |

This checklist is copyrighted by the Equator Network under the Creative Commons Attribution 3.0 Unported (CC BY 3.0) license.

1. Skrivankova VW, Richmond RC, Woolf BAR, Yarmolinsky J, Davies NM, Swanson SA, et al. Strengthening the Reporting of Observational Studies in Epidemiology using Mendelian Randomization (STROBE-MR) Statement. JAMA. 2021;under review.

2. Skrivankova VW, Richmond RC, Woolf BAR, Davies NM, Swanson SA, VanderWeele TJ, et al. Strengthening the Reporting of Observational Studies in Epidemiology using Mendelian Randomisation (STROBE-MR): Explanation and Elaboration. BMJ. 2021;375:n2233.

## Data Sources
### T2D and glycemic traits

We obtained the genetic instruments of T2D of European descent from the Diabetes Meta-Analysis of Trans-Ethics associations studies (DIAMANTE) Consortium (80,154 cases and 853,816 controls) [1]. T2D cases were defined as FG≥7mmol/l, or 2-hour glucose (2hGlu)≥11.1mmol/l, glycated hemoglobin (HbA1c)≥6.5%, on T2D treatment etc., while controls were defined as participants not fulfilling the case definition or not reporting as T2D [1]. Genetic associations were estimated using a linear mixed model with adjustment for age, sex, and additional study-specific covariates [1].

The GWAS summary statistics of glycemic traits, including FG (mmol/l, $n$ = 209,605), 2hGlu (mmol/l, $n$ = 64,469), FI (log pmol/l, $n$ = 158,550), and HbA1c (%, $n$ =149,006), were from the Meta-Analyses of Glucose and Insulin-related traits Consortium (MAGIC) in participants of European ancestry [2]. Participants who had type 1 diabetes, or T2D, diabetes-related medication, FG≥7mmol/l, or 2hGlu≥11.1mmol/l, HbA1c≥6.5% were excluded [2]. Genetic associations were estimated from a linear mixed model with adjustment for age, sex, study-specific covariates, principal components and body mass index (BMI) (except for HbA1c) [2]. The original GWAS showed the collider bias from adjusting for BMI had little impact on the associations of the signals (**Table S3**) [2].

### Iron homeostasis biomarkers

We extracted the genetic instruments of iron homeostasis biomarkers including ferritin (standard deviation (SD)) ($n$ = 246,139), serum iron (SD, $n$ = 163,511), TIBC (SD, $n$ = 135,430), and TSAT (SD, $n$ = 131,471) from GWAS summary statistics of participants of European ancestry from deCODE genetics project (Iceland), INTERVAL study (UK), and Danish Blood Donor Study (Denmark) [3]. The iron biomarkers were inverse normal

transformed (separately for each sex), and a generalized additive model was used to obtain age-adjusted biomarker values, and ditto for INTERVAL (additional adjusting for menopausal status, ABO blood group, BMI, smoking, alcohol, and iron supplementation). These adjusted values were then used to obtain genetic association with biomarkers. BOLT-LMM were taken into account population stratification and relatedness. Meta-analyses were used to combine the summary statistics from the three cohorts. (**Table S3**) [3].

**Liver steatosis, liver cirrhosis, ALT, and PDFF**

We fetched the genetic instruments of steatosis (log odds, 9491 cases and 876,210 controls) from GWAS summary statistics of participants of European ancestry from UK Biobank (UKB, 5921 cases), Icelandic deCODE genetics study (deCODE, 785 cases), FinnGen (651 cases), and USA Intermountain dataset (INTERMOUNTAIN, 2,134 cases), and liver cirrhosis (log odds, 4809 cases and 967,898 controls) from UKB (2301 cases), deCODE (691 cases), FinnGen (1,425 cases), and INTERMOUNTAIN (392 cases) [4]. Steatosis cases were identified by the diagnostic codes of ICD-10 (K76.0) relating to Fatty (change of) liver, not elsewhere classified in electronic health records, while all-cause liver cirrhosis was defined by the diagnostic codes of ICD-10 (K70.2, K70.3, K70.4, K74.0, K74.1, K74.2, K74.6, K76.6 and KI85) related to cirrhosis and fibrosis [4]. Genetic associations were estimated from logistic regressions with adjustment for age, sex and additionally adjusted for population stratification (relied on 40 principal components) in UKB, county of birth, blood sample availability for the individual and an indicator function for the overlap of the lifetime of the individual with the time span of the phenotype collection in deCODE, 10 principal components, FinnGen 1 or 2 chip or legacy genotyping batch in FinnGen, and first 10 principal components in INTERMOUNTAIN (**Table S3**) [4].

The genetic instruments of ALT (SD, *n*=344,136) and PDFF (SD, *n*=36,116) were obtained from the GWAS summary statistics of participants of European ancestry from UKB [4]. The genetic associations of ALT were estimated by linear mixed model with inverse-rank normal transformation and adjustments for age, sex, age*sex, $age^2$, $age^2$*sex, and first 10 principal components, which we obtained from Integrative Epidemiology Unit (IEU) GWAS database (id: ukb-d-30620_irnt) [5]. PDFF scores were derived from the raw abdominal Magnetic resonance (MR) images, which utilized two acquisition technologies including gradient multiecho (GRE, *n*=8,448) and iterative decomposition of water and fat with echo asymmetry and least-squares estimation (IDEAL, *n*=27,668), and they were computed by a three-point Dixon method (GRE) and a specified signal model (IDEAL) [4]. The corresponding PDFF scores (%) between the IDEAL and GRE acquisition were then evaluated by a linear model [4]. The genetic associations were assessed using a linear mixed model adjusted for sex, year of birth, age at measurement (if available), and BMI, and standardized for being normal distributed based on the first three covariates (**Table S3**) [4].

**Liver iron**

The genetic instruments of MR imaging (MRI)-derived liver iron (SD, *n*=32,858) were obtained from the GWAS summary statistics of participants of European ancestry from UKB [6]. All abdominal scans were analyzed by two acquisitions including the Dixon protocol and a single-slice multi-echo acquisition sequence [6]. Through an automated fat-water swap detection and correction, the Dixon data for each participant were consolidated into a unified 3D volume [6]. The liver iron concentration (mg/g) were estimated by the R2* values [6]. Participants with discordant sex information were removed. The genetic associations were estimated by a generalized linear mixed model adjusted for age at imaging visit, $age^2$, sex,

imaging centre, scan date, scan time, and genotyping batch, and genetic relatedness derived from genotyped SNPs as a random effect [6].

References


1. Mahajan A, Spracklen CN, Zhang W, Ng MCY, Petty LE, Kitajima H, Yu GZ, Rüeger S, Speidel L, Kim YJ *et al*: **Multi-ancestry genetic study of type 2 diabetes highlights the power of diverse populations for discovery and translation**. *Nat Genet* 2022, **54**(5):560-572.
2. Chen J, Spracklen CN, Marenne G, Varshney A, Corbin LJ, Luan J, Willems SM, Wu Y, Zhang X, Horikoshi M *et al*: **The trans-ancestral genomic architecture of glycemic traits**. *Nat Genet* 2021, **53**(6):840-860.
3. Bell S, Rigas AS, Magnusson MK, Ferkingstad E, Allara E, Bjornsdottir G, Ramond A, Sørensen E, Halldorsson GH, Paul DS *et al*: **A genome-wide meta-analysis yields 46 new loci associating with biomarkers of iron homeostasis**. *Commun Biol* 2021, **4**(1):156.
4. Sveinbjornsson G, Ulfarsson MO, Thorolfsdottir RB, Jonsson BA, Einarsson E, Gunnlaugsson G, Rognvaldsson S, Arnar DO, Baldvinsson M, Bjarnason RG *et al*: **Multiomics study of nonalcoholic fatty liver disease**. *Nat Genet* 2022, **54**(11):1652-1663.
5. Elsworth B, Lyon M, Alexander T, Liu Y, Matthews P, Hallett J, Bates P, Palmer T, Haberland V, Smith GD *et al*: **The MRC IEU OpenGWAS data infrastructure**. *bioRxiv* 2020:2020.2008.2010.244293.
6. Liu Y, Basty N, Whitcher B, Bell JD, Sorokin EP, van Bruggen N, Thomas EL, Cule M: **Genetic architecture of 11 organ traits derived from abdominal MRI using deep learning**. *Elife* 2021, **10**:e65554.
