# Supplementary material for: Do iron homeostasis biomarkers mediate the associations of liability to type 2 diabetes and glycemic traits in liver steatosis and cirrhosis: a two-step Mendelian randomization study
Source: BMC Med. 2024 Jun 26;22:270. doi: 10.1186/s12916-024-03486-w (PMC11210020; doi:10.1186/s12916-024-03486-w)
Supplement: Supplementary file 3 — Additional file 3: Fig. S1 The associations of liability to type 2 diabetes and glycemic traits in liver steatosis and liver cirrhosis using meta-analysis of Inverse-variance weighting and MR-PRESSO (outlier-corrected). Fig. S2 The associations of HbA1c signal classifications in liver steatosis, ferritin, and liver iron using Mendelian randomization. Fig. S3 (a) Scatter plots and (b) forest plot of fasting glucose and liver steatosis in UK Biobank, deCODE, FinnGen, and INTERMOUNTAIN. Fig. S4 The associations of iron homeostasis biomarkers in liver steatosis and liver cirrhosis using meta-analysis of Inverse-variance weighting and MR-PRESSO (outlier-corrected). Fig. S5 The association of liability to type 2 diabetes and glycemic traits in liver iron (MRI) using Inverse-variance weighting. Fig. S6 The associations of fasting insulin, ferritin, and liver iron in liver steatosis with mediators and exposures adjustment using multivariable Mendelian randomization analysis. [file 12916_2024_3486_MOESM3_ESM.pdf]

Supplementary Figures

Do iron homeostasis biomarkers mediate the associations of liability to type 2 diabetes and glycemic traits in liver steatosis and cirrhosis: a two-step Mendelian randomization study

Ying Liang[1], Shan Luo[1], Steven Bell[2,3], Jacky Man Yuen Mo[1], Baoting He[1], Yangzhong Zhou[4], Xiaoyin Bai[5], Shiu Lun Au Yeung[1*]

[1] School of Public Health, LKS Faculty of Medicine, The University of Hong Kong, Hong Kong SAR, China

[2] Precision Breast Cancer Institute, Department of Oncology, University of Cambridge, Cambridge, UK

[3] Cancer Research UK Cambridge Institute, Li Ka Shing Centre, University of Cambridge, Cambridge, UK

[4] Department of Rheumatology, Peking Union Medical College Hospital, National Clinical Research Center for Dermatologic and Immunologic Diseases (NCRC-DID), Beijing 100730, China

[5] Department of Gastroenterology, Peking Union Medical College Hospital, Peking Union Medical College and Chinese Academy of Medical Sciences, Beijing 100730, China

***Corresponding author**

Legends of these supplementary figures

Page 1: **Fig. S1** The associations of liability to type 2 diabetes and glycemic traits in liver steatosis and liver cirrhosis using meta-analysis of Inverse-variance weighting and MR-PRESSO (outlier-corrected).

Page 2: **Fig. S2** The associations of HbA1c signal classifications in liver steatosis, ferritin, and liver iron using Mendelian randomization.

Page 3: **Fig. S3** (a) Scatter plots and (b) forest plot of fasting glucose and liver steatosis in UK Biobank, deCODE, FinnGen, and INTERMOUNTAIN.

Page 4: **Fig. S4** The associations of iron homeostasis biomarkers in liver steatosis and liver cirrhosis using meta-analysis of Inverse-variance weighting and MR-PRESSO (outlier-corrected).

Page 5: **Fig. S5** The association of liability to type 2 diabetes and glycemic traits in liver iron (MRI) using Inverse-variance weighting.

Page 6: **Fig. S6** The associations of fasting insulin, ferritin, and liver iron in liver steatosis with mediators and exposures adjustment using multivariable Mendelian randomization analysis.

A

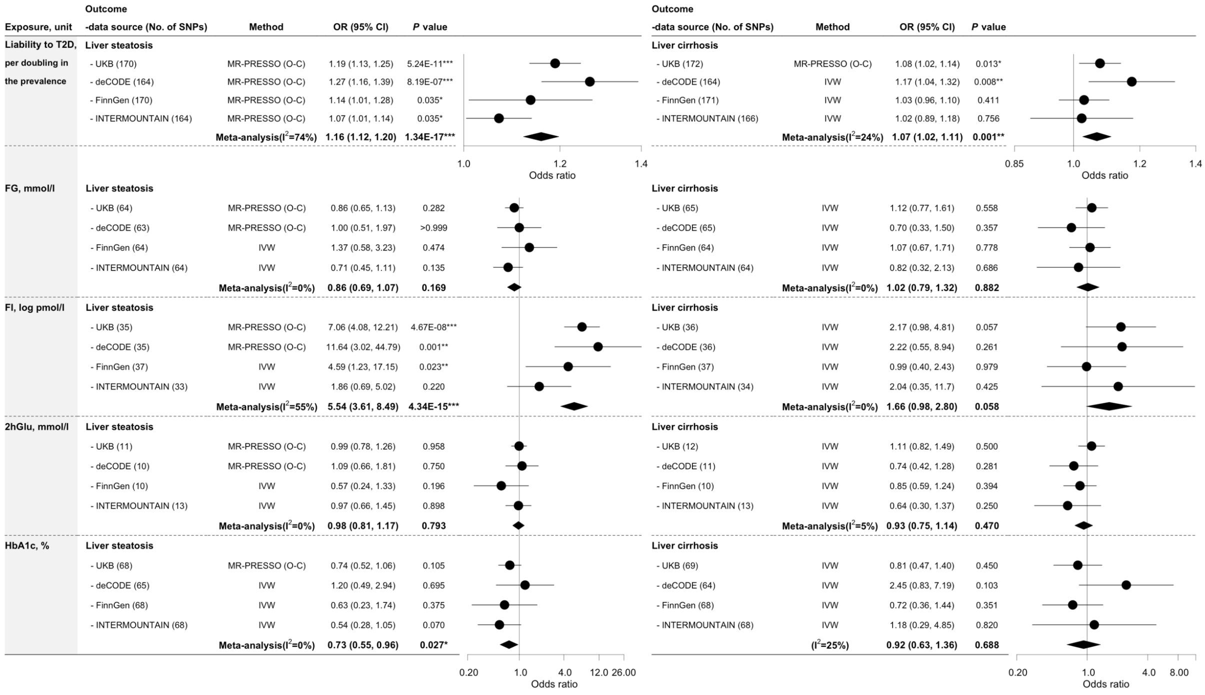

Exposure, unit	Outcome -data source (No. of SNPs)	Method	OR (95% CI)	*P* value	Outcome -data source (No. of SNPs)	Method	OR (95% CI)	*P* value
**Liability to T2D, per doubling in the prevalence**	**Liver steatosis**				**Liver cirrhosis**			
	- UKB (170)	MR-PRESSO (O-C)	1.19 (1.13, 1.25)	5.24E-11***	- UKB (172)	MR-PRESSO (O-C)	1.08 (1.02, 1.14)	0.013*
	- deCODE (164)	MR-PRESSO (O-C)	1.27 (1.16, 1.39)	8.19E-07***	- deCODE (164)	IVW	1.17 (1.04, 1.32)	0.008**
	- FinnGen (170)	MR-PRESSO (O-C)	1.14 (1.01, 1.28)	0.035*	- FinnGen (171)	IVW	1.03 (0.96, 1.10)	0.411
	- INTERMOUNTAIN (164)	MR-PRESSO (O-C)	1.07 (1.01, 1.14)	0.035*	- INTERMOUNTAIN (166)	IVW	1.02 (0.89, 1.18)	0.756
		**Meta-analysis(I^2=74%)**	**1.16 (1.12, 1.20)**	**1.34E-17*** **		**Meta-analysis(I^2=24%)**	**1.07 (1.02, 1.11)**	**0.001** **
**FG, mmol/l**	**Liver steatosis**				**Liver cirrhosis**			
	- UKB (64)	MR-PRESSO (O-C)	0.86 (0.65, 1.13)	0.282	- UKB (65)	IVW	1.12 (0.77, 1.61)	0.558
	- deCODE (63)	MR-PRESSO (O-C)	1.00 (0.51, 1.97)	>0.999	- deCODE (65)	IVW	0.70 (0.33, 1.50)	0.357
	- FinnGen (64)	IVW	1.37 (0.58, 3.23)	0.474	- FinnGen (64)	IVW	1.07 (0.67, 1.71)	0.778
	- INTERMOUNTAIN (64)	IVW	0.71 (0.45, 1.11)	0.135	- INTERMOUNTAIN (64)	IVW	0.82 (0.32, 2.13)	0.686
		**Meta-analysis(I^2=0%)**	**0.86 (0.69, 1.07)**	**0.169**		**Meta-analysis(I^2=0%)**	**1.02 (0.79, 1.32)**	**0.882**
**FI, log pmol/l**	**Liver steatosis**				**Liver cirrhosis**			
	- UKB (35)	MR-PRESSO (O-C)	7.06 (4.08, 12.21)	4.67E-08***	- UKB (36)	IVW	2.17 (0.98, 4.81)	0.057
	- deCODE (35)	MR-PRESSO (O-C)	11.64 (3.02, 44.79)	0.001**	- deCODE (36)	IVW	2.22 (0.55, 8.94)	0.261
	- FinnGen (37)	IVW	4.59 (1.23, 17.15)	0.023**	- FinnGen (37)	IVW	0.99 (0.40, 2.43)	0.979
	- INTERMOUNTAIN (33)	IVW	1.86 (0.69, 5.02)	0.220	- INTERMOUNTAIN (34)	IVW	2.04 (0.35, 11.7)	0.425
		**Meta-analysis(I^2=55%)**	**5.54 (3.61, 8.49)**	**4.34E-15*** **		**Meta-analysis(I^2=0%)**	**1.66 (0.98, 2.80)**	**0.058**
**2hGlu, mmol/l**	**Liver steatosis**				**Liver cirrhosis**			
	- UKB (11)	MR-PRESSO (O-C)	0.99 (0.78, 1.26)	0.958	- UKB (12)	IVW	1.11 (0.82, 1.49)	0.500
	- deCODE (10)	MR-PRESSO (O-C)	1.09 (0.66, 1.81)	0.750	- deCODE (11)	IVW	0.74 (0.42, 1.28)	0.281
	- FinnGen (10)	IVW	0.57 (0.24, 1.33)	0.196	- FinnGen (10)	IVW	0.85 (0.59, 1.24)	0.394
	- INTERMOUNTAIN (13)	IVW	0.97 (0.66, 1.45)	0.898	- INTERMOUNTAIN (13)	IVW	0.64 (0.30, 1.37)	0.250
		**Meta-analysis(I^2=0%)**	**0.98 (0.81, 1.17)**	**0.793**		**Meta-analysis(I^2=5%)**	**0.93 (0.75, 1.14)**	**0.470**
**HbA1c, %**	**Liver steatosis**				**Liver cirrhosis**			
	- UKB (68)	MR-PRESSO (O-C)	0.74 (0.52, 1.06)	0.105	- UKB (69)	IVW	0.81 (0.47, 1.40)	0.450
	- deCODE (65)	IVW	1.20 (0.49, 2.94)	0.695	- deCODE (64)	IVW	2.45 (0.83, 7.19)	0.103
	- FinnGen (68)	IVW	0.63 (0.23, 1.74)	0.375	- FinnGen (68)	IVW	0.72 (0.36, 1.44)	0.351
	- INTERMOUNTAIN (68)	IVW	0.54 (0.28, 1.05)	0.070	- INTERMOUNTAIN (68)	IVW	1.18 (0.29, 4.85)	0.820
		**Meta-analysis(I^2=0%)**	**0.73 (0.55, 0.96)**	**0.027***		**(I^2=25%)**	**0.92 (0.63, 1.36)**	**0.688**

B

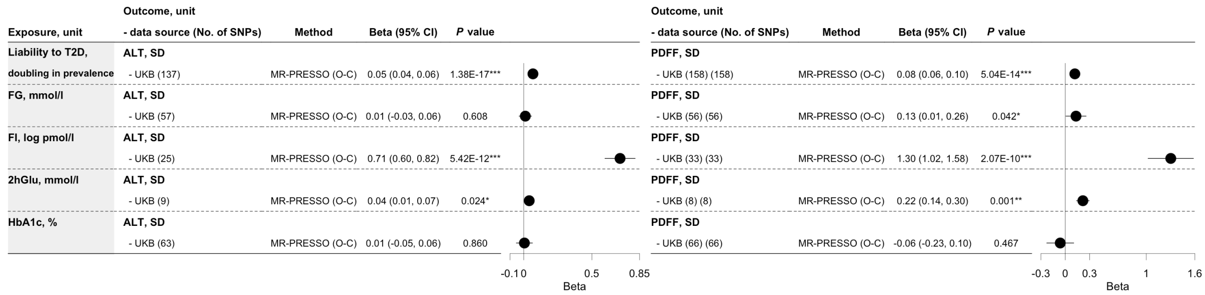

Exposure, unit	Outcome, unit - data source (No. of SNPs)	Method	Beta (95% CI)	*P* value	Outcome, unit - data source (No. of SNPs)	Method	Beta (95% CI)	*P* value
**Liability to T2D, doubling in prevalence**	**ALT, SD**				**PDFF, SD**			
	- UKB (137)	MR-PRESSO (O-C)	0.05 (0.04, 0.06)	1.38E-17***	- UKB (158) (158)	MR-PRESSO (O-C)	0.08 (0.06, 0.10)	5.04E-14***
**FG, mmol/l**	**ALT, SD**				**PDFF, SD**			
	- UKB (57)	MR-PRESSO (O-C)	0.01 (-0.03, 0.06)	0.608	- UKB (56) (56)	MR-PRESSO (O-C)	0.13 (0.01, 0.26)	0.042*
**FI, log pmol/l**	**ALT, SD**				**PDFF, SD**			
	- UKB (25)	MR-PRESSO (O-C)	0.71 (0.60, 0.82)	5.42E-12***	- UKB (33) (33)	MR-PRESSO (O-C)	1.30 (1.02, 1.58)	2.07E-10***
**2hGlu, mmol/l**	**ALT, SD**				**PDFF, SD**			
	- UKB (9)	MR-PRESSO (O-C)	0.04 (0.01, 0.07)	0.024*	- UKB (8) (8)	MR-PRESSO (O-C)	0.22 (0.14, 0.30)	0.001**
**HbA1c, %**	**ALT, SD**				**PDFF, SD**			
	- UKB (63)	MR-PRESSO (O-C)	0.01 (-0.05, 0.06)	0.860	- UKB (66) (66)	MR-PRESSO (O-C)	-0.06 (-0.23, 0.10)	0.467

Fig. S1 The associations of liability to type 2 diabetes and glycemic traits in liver steatosis and liver cirrhosis using meta-analysis of Inverse-variance weighting and MR-PRESSO (outlier-corrected). (A) The associations of liability to type 2 diabetes and glycemic traits in liver steatosis. (B) The associations of liability to type 2 diabetes and glycemic traits in liver cirrhosis. Liability to T2D, liability to type 2 diabetes; FG, fasting glucose; FI, fasting insulin; 2hGlu, two-hour glucose; HbA1c, hemoglobin A1c; No. of SNPs, number of single nucleotide polymorphisms; IVW, Inverse-variance weighting; MR-PRESSO (O-C), Mendelian Randomization Pleiotropy RESidual Sum and Outlier (Outlier-corrected); I^2, degree of heterogeneity; OR, odds ratio; 95% CI, 95% confidence interval. **P* value <0.05, ***P* value <0.01, ****P* value <0.001.

A

Exposure	Outcome	Method	OR (95% CI)	*P* value
HbA1c	Liver steatosis	Meta-analysis of IVW		
Signal classification	(UKB, deCODE, FinnGen,			
- Glycemic	INTERMOUNTAIN)		0.83 (0.39, 1.78)	0.635
- Iron			0.87 (0.24, 3.09)	0.828
- Mature RBC			0.62 (0.36, 1.06)	0.083
- Reticulocyte			0.88 (0.44, 1.76)	0.720
- Unknown			0.89 (0.40, 1.96)	0.769
**All SNPs**			**0.70 (0.51, 0.96)**	**0.027***

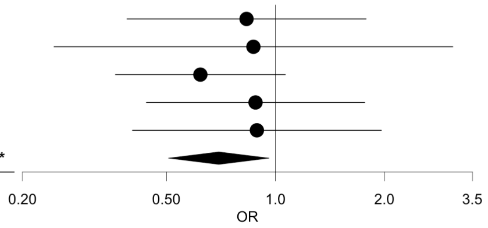

B

Exposure	Outcome	Method	No. of SNPs	Beta (95% CI)	*P* value
HbA1c, %	Ferritin, SD	IVW			
Signal classification					
- Glycemic			14	0.04 (-0.22, 0.31)	0.755
- Iron			3	-1.63 (-3.13, -0.13)	0.033*
- Mature RBC			22	-0.35 (-0.53, -0.17)	1.80E-04***
- Reticulocyte			17	-0.47 (-0.76, -0.19)	0.001**
- Unknown			14	-0.12 (-0.44, 0.20)	0.467
**All SNPs**			**70**	**-0.31 (-0.46, -0.17)**	**2.33E-05*** **
Signal classification	Liver iron, SD	IVW			
- Glycemic			15	0.08 (-0.26, 0.42)	0.659
- Iron			3	-3.36 (-6.24, -0.47)	0.023*
- Mature RBC			22	-0.43 (-0.66, -0.19)	3.76E-04***
- Reticulocyte			18	0.01 (-0.3, 0.32)	0.961
- Unknown			14	-0.26 (-0.68, 0.16)	0.224
**All SNPs**			**72**	**-0.34 (-0.56, -0.12)**	**0.003*** *

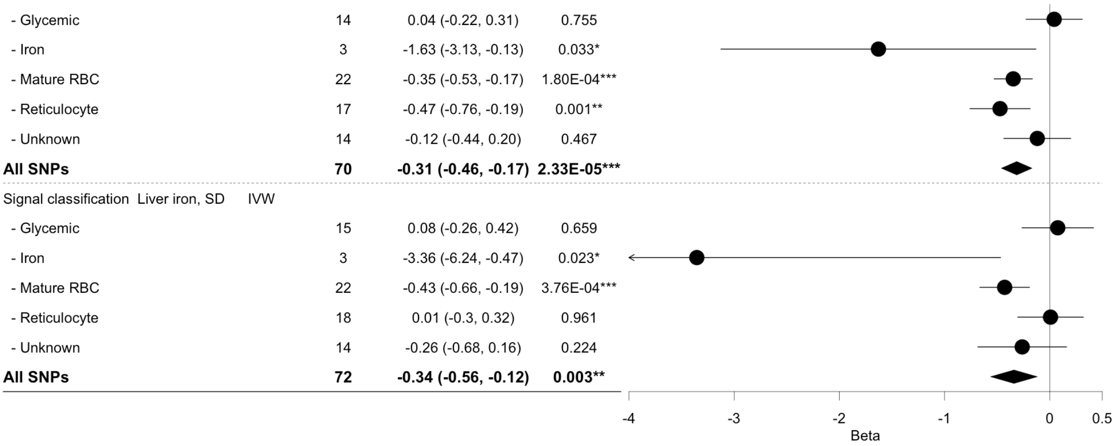

Fig. S2 The associations of HbA1c signal classifications in liver steatosis, ferritin, and liver iron using Mendelian randomization. (A) The associations of HbA1c signal classifications in liver steatosis; (B) The associations of HbA1c signal classifications in ferritin and liver iron. HbA1c, hemoglobin A1c; IVW, Inverse-variance weighting; No. of SNPs, number of single nucleotide polymorphisms; Glycemic, probability of the variant in glycemic class, included fasting insulin, 2hr-glucose, and fasting glucose; Reticulocyte, probability of the variant in reticulocyte class, included reticulocyte count, reticulocyte fraction of red cells, immature fraction of reticulocytes, high light scatter reticulocyte count, and high light scatter reticulocyte percentage of red cells; Mature RBC, probability of the variant in mature red blood cell class, included Red blood cell count, mean corpuscular volume, hematocrit, mean corpuscular hemoglobin, mean corpuscular hemoglobin concentration, hemoglobin concentration, and red cell distribution width; Iron, probability of the variant in iron class, included ferritin, transferrin, serum iron, and transferrin saturation; Unknown, probability of the variant in unknown class; OR, odds ratio; 95% CI, 95% confidence interval. **P* value <0.05, ***P* value <0.01, ****P* value <0.001.

A

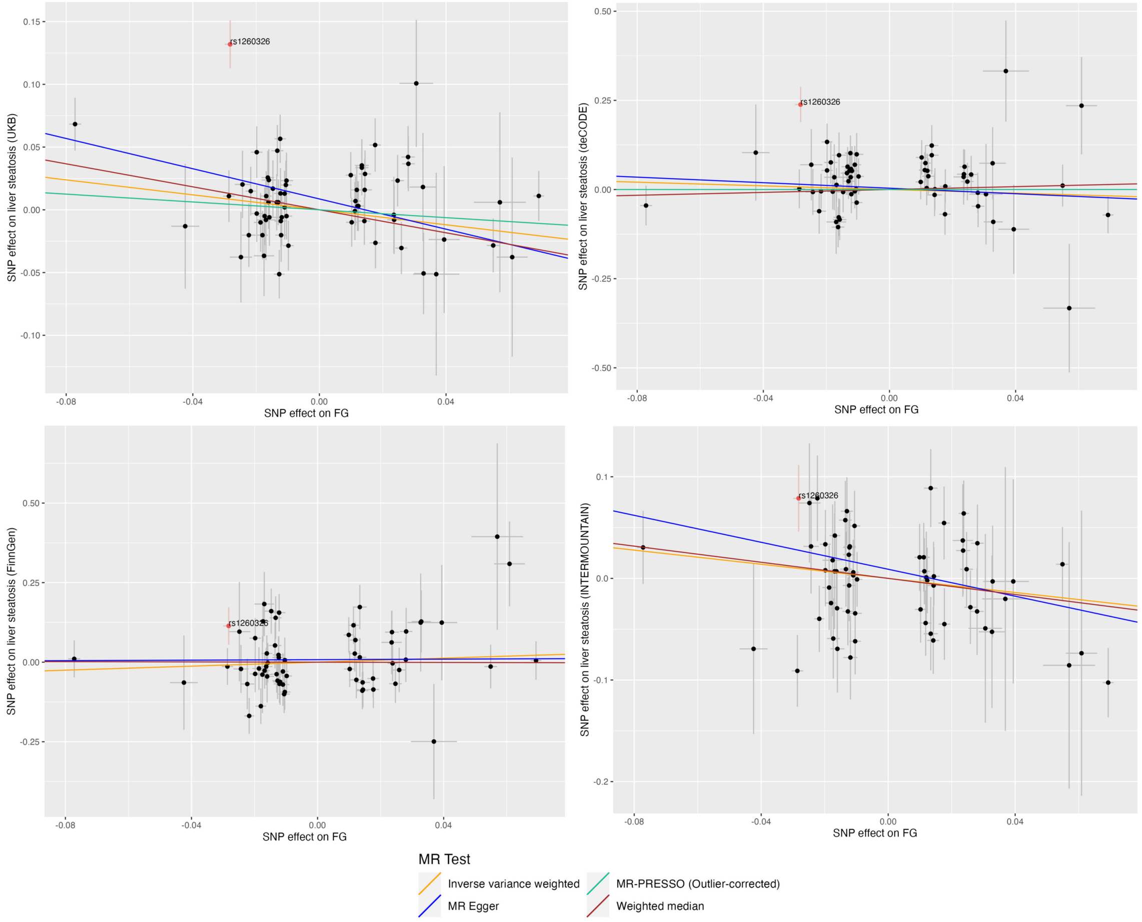

B

Exposure	Outcome (data source)	Analysis	Method	No. of SNPs	OR (95% CI)	*P* value
FG, mmol/l	Liver steatosis					
	- UKB	All SNPs	IVW	65	0.74 (0.53, 1.04)	0.082
		Excl. rs1260326	IVW	64	0.86 (0.65, 1.13)	0.278
	- deCODE	All SNPs	IVW	64	0.77 (0.36, 1.64)	0.505
		Excl. rs1260326	IVW	63	1.00 (0.51, 1.97)	>0.999
	- FinnGen	All SNPs	IVW	64	1.37 (0.58, 3.23)	0.474
		Excl. rs1260326	IVW	63	1.55 (0.66, 3.66)	0.315
	- INTERMOUNTAIN	All SNPs	IVW	64	0.71 (0.45, 1.11)	0.135
		Excl. rs1260326	IVW	63	0.76 (0.48, 1.20)	0.243
		All SNPs	Meta-analysis		0.77 (0.60, 0.99)	0.038*
		Excl. rs1260326	of IVW		0.88 (0.71, 1.09)	0.251

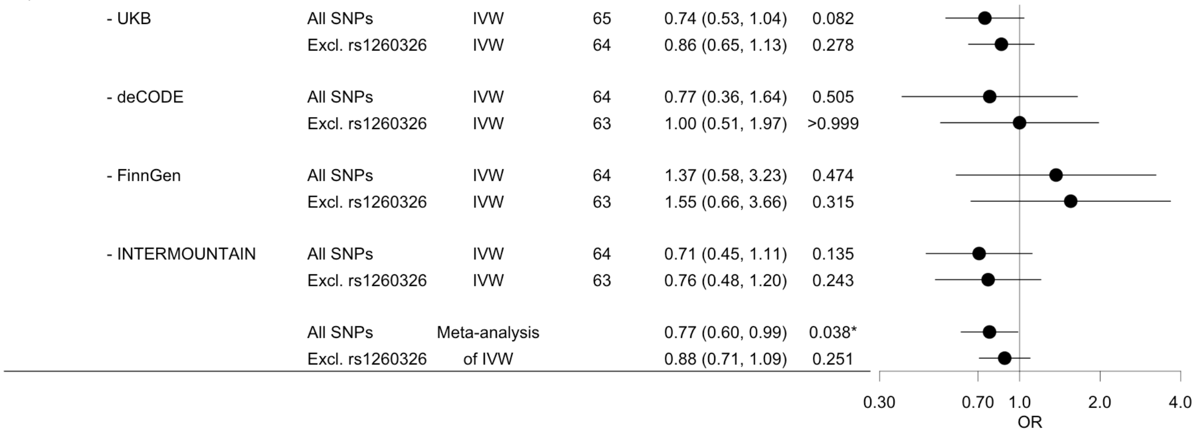

Fig. S3 (A) Scatter plots and (B) forest plot of fasting glucose and liver steatosis in UK Biobank, deCODE, FinnGen, and INTERMOUNTAIN. SNP, single nucleotide polymorphism; FG, fasting glucose. In (a), The highlighted SNP, rs1260326 (*GCKR*), was removed in MR-PRESSO (Outlier-corrected) in UK Biobank and deCODE. In (B), excl. rs1260326 means with the exclusion of rs1260326 (*GCKR*).

A

Exposure, unit	Outcome -data source(No. of SNPs)	Method	OR (95% CI)	*P* value	Outcome -data source(No. of SNPs)	Method	OR (95% CI)	*P* value
**Ferritin, SD**	**Liver steatosis**				**Liver cirrhosis**			
	- UKB (57)	MR-PRESSO (O-C)	1.38 (1.16, 1.63)	4.50E-04***	- UKB (56)	MR-PRESSO (O-C)	0.80 (0.59, 1.08)	0.151
	- deCODE (49)	MR-PRESSO (O-C)	1.04 (0.67, 1.63)	0.853	- deCODE (51)	IVW	1.28 (0.75, 2.19)	0.369
	- FinnGen (55)	IVW	1.17 (0.69, 1.98)	0.568	- FinnGen (54)	MR-PRESSO (O-C)	1.11 (0.74, 1.68)	0.608
	- INTERMOUNTAIN (58)	IVW	1.02 (0.77, 1.34)	0.914	- INTERMOUNTAIN (58)	IVW	1.77 (0.84, 3.74)	0.132
		**Meta-analysis(I^2=27%)**	**1.24 (1.09, 1.42)**	**0.001****		**(I^2=45%)**	**1.00 (0.81, 1.24)**	**0.972**
**Serum iron, SD**	**Liver steatosis**				**Liver cirrhosis**			
	- UKB (25)	IVW	1.20 (0.98, 1.46)	0.075	- UKB (25)	MR-PRESSO (O-C)	1.06 (0.81, 1.39)	0.683
	- deCODE (26)	IVW	1.17 (0.74, 1.86)	0.495	- deCODE (26)	IVW	1.23 (0.65, 2.35)	0.524
	- FinnGen (22)	IVW	1.06 (0.56, 1.99)	0.865	- FinnGen (22)	IVW	0.85 (0.55, 1.32)	0.474
	- INTERMOUNTAIN (27)	IVW	1.05 (0.81, 1.36)	0.732	- INTERMOUNTAIN (27)	IVW	1.62 (0.88, 2.97)	0.123
		**Meta-analysis(I^2=0%)**	**1.14 (0.98, 1.32)**	**0.080**		**(I^2=0%)**	**1.07 (0.88, 1.32)**	**0.489**
**TIBC, SD**	**Liver steatosis**				**Liver cirrhosis**			
	- UKB (30)	IVW	0.95 (0.84, 1.07)	0.382	- UKB (29)	MR-PRESSO (O-C)	1.00 (0.79, 1.28)	0.981
	- deCODE (27)	IVW	0.79 (0.59, 1.07)	0.127	- deCODE (26)	IVW	0.93 (0.63, 1.37)	0.701
	- FinnGen (27)	IVW	1.11 (0.75, 1.64)	0.610	- FinnGen (27)	IVW	0.98 (0.72, 1.34)	0.893
	- INTERMOUNTAIN (30)	IVW	1.22 (0.99, 1.51)	0.062	- INTERMOUNTAIN (31)	IVW	1.10 (0.72, 1.67)	0.665
		**(I^2=56%)**	**0.99 (0.90, 1.09)**	**0.820**		**(I^2=0%)**	**1.00 (0.85, 1.17)**	**0.964**
**TSAT, SD**	**Liver steatosis**				**Liver cirrhosis**			
	- UKB (24)	IVW	1.08 (0.95, 1.23)	0.213	- UKB (24)	MR-PRESSO (O-C)	0.98 (0.80, 1.21)	0.869
	- deCODE (22)	IVW	1.19 (0.84, 1.68)	0.322	- deCODE (23)	IVW	1.10 (0.72, 1.68)	0.657
	- FinnGen (22)	IVW	1.03 (0.70, 1.50)	0.892	- FinnGen (22)	IVW	1.00 (0.77, 1.30)	0.985
	- INTERMOUNTAIN (26)	IVW	0.91 (0.72, 1.16)	0.446	- INTERMOUNTAIN (26)	IVW	1.09 (0.66, 1.79)	0.748
		**(I^2=0%)**	**1.05 (0.95, 1.17)**	**0.312**		**(I^2=0%)**	**1.01 (0.87, 1.17)**	**0.89**

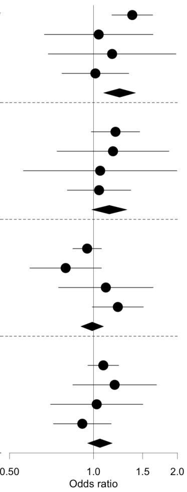

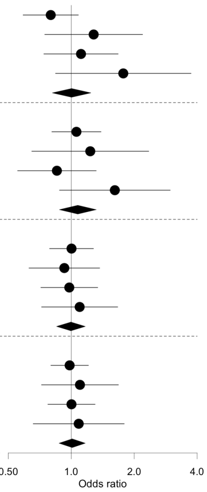

B

Exposure, unit	Outcome, unit - data source (No. of SNPs)	Method	Beta (95% CI)	*P* value	Outcome, unit - data source (No. of SNPs)	Method	Beta (95% CI)	*P* value
**Ferritin, SD**	**ALT, SD**				**PDFF, SD**			
	- UKB (53)	MR-PRESSO (O-C)	0.02 (-0.01, 0.06)	0.164	- UKBB (55)	MR-PRESSO (O-C)	-5.3E-04 (-0.07, 0.07)	0.988
**Serum iron, SD**	**ALT, SD**				**PDFF, SD**			
	- UKB (24)	MR-PRESSO (O-C)	0.01 (-0.02, 0.04)	0.475	- UKBB (25)	MR-PRESSO (O-C)	0.04 (-0.04, 0.13)	0.314
**TIBC, SD**	**ALT, SD**				**PDFF, SD**			
	- UKB (23)	MR-PRESSO (O-C)	-0.01 (-0.03, 0.01)	0.369	- UKBB (28)	MR-PRESSO (O-C)	0.03 (-0.04, 0.10)	0.394
**TSAT, SD**	**ALT, SD**				**PDFF, SD**			
	- UKB (23)	MR-PRESSO (O-C)	0.02 (2.6E-03, 0.04)	0.038*	- UKBB (23)	MR-PRESSO (O-C)	0.03 (-0.03, 0.09)	0.347

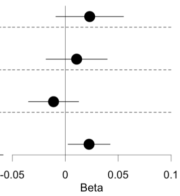

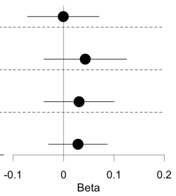

Fig. S4 The associations of iron homeostasis biomarkers in liver steatosis and liver cirrhosis using meta-analysis of Inverse-variance weighting and MR-PRESSO (outlier-corrected). (A) The associations of iron homeostasis biomarkers in liver steatosis; (B) The associations of iron homeostasis biomarkers in liver cirrhosis. Liver steatosis, log odds (*n* = 885,701 including 9,491 cases and 876,210 controls) included UKB (5,921 cases), deCODE (785 cases), FinnGen (651 cases), and INTERMOUNTAIN (2,134 cases). Liver cirrhosis, log odds (*n* = 972,707 including 4,809 cases and 967,898 controls) included UKB (2,301 cases), deCODE (691 cases), FinnGen (1,425 cases), and INTERMOUNTAIN (392 cases). TIBC, total iron-binding capacity; TSAT, transferrin saturation; No. of SNPs, number of single nucleotide polymorphisms; IVW, Inverse-variance weighting; MR-PRESSO (O-C), Mendelian Randomization Pleiotropy RESidual Sum and Outlier (Outlier-corrected); I^2, degree of heterogeneity; OR, odds ratio; 95% CI, 95% confidence interval. **P* value <0.05, ***P* value <0.01, ****P* value <0.001.

Exposure, unit	Outcome, unit - data source (No. of SNPs)	Method	I^2	Beta (95% CI)	*P* value
**Liability to T2D**	**Liver iron (MRI), SD**				
	- UKB (178)	IVW	27%	-0.01 (-0.03, 0.01)	0.238
**FG, mmol/l**	**Liver iron (MRI), SD**				
	- UKB (66)	IVW	24%	0.07 (-0.04, 0.17)	0.230
**FI, log pmol/l**	**Liver iron (MRI), SD**				
	- UKB (37)	IVW	37%	0.03 (-0.19, 0.25)	0.797
**2hGlu, mmol/l**	**Liver iron (MRI), SD**				
	- UKB (12)	IVW	70%	1.8E-03 (-0.13, 0.13)	0.978
**HbA1c, %**	**Liver iron (MRI), SD**				
	- UKB (72)	IVW	66%	-0.34 (-0.56, -0.12)	0.003**

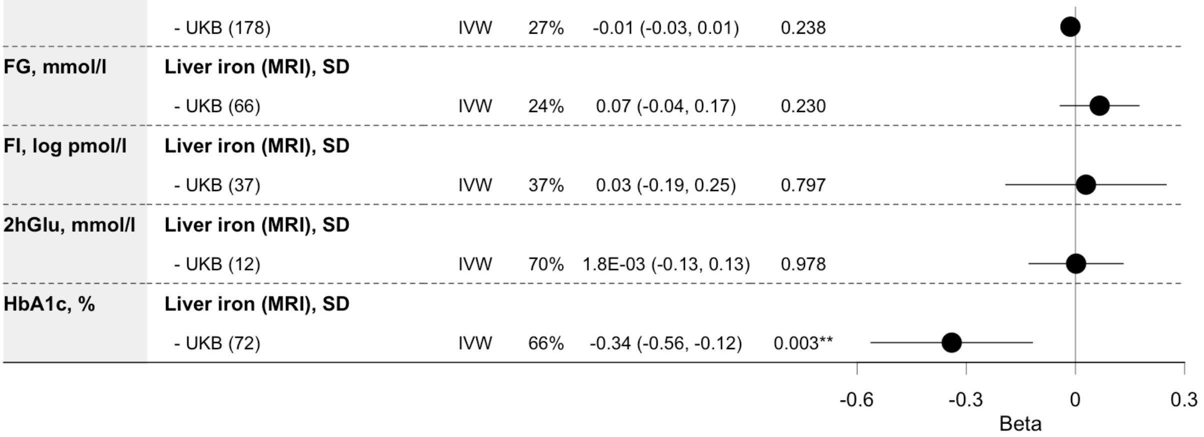

Fig. S5 The associations of liability to type 2 diabetes and glycemic traits in liver iron (MRI) using Inverse-variance weighting. Liability to T2D, liability to type 2 diabetes, per doubling in prevalence; FG, fasting glucose; FI, fasting insulin; 2hGlu, two-hour glucose; HbA1c, hemoglobin A1c; No. of SNPs, number of single nucleotide polymorphisms; IVW, Inverse-variance weighting; I^2, degree of heterogeneity; 95% CI, 95% confidence interval. **P* value <0.05, ***P* value <0.01, ****P* value <0.001.

A

Exposure, unit	Outcome -data source (No. of SNPs)	Method	OR (95% CI)	*P* value	Conditional F-statistic
**FI, log pmol/l**	**Liver steatosis**	**Adjusted for ferritin**			
	- UKB (34)	MVMR IVW	2.91 (1.62, 5.26)	3.82E-04***	50
	- deCODE (34)	MVMR IVW	5.60 (1.37, 22.90)	0.016*	50
	- FinnGen (35)	MVMR IVW	5.43 (1.47, 20.07)	0.011*	49
	- INTERMOUNTAIN (31)	MVMR IVW	1.84 (0.81, 4.16)	0.143	50
		**Meta-analysis(I^2=0%)**	**2.92 (1.90, 4.47)**	**9.49E-07*** **	

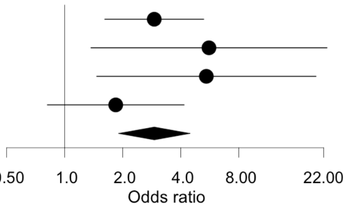

B

Exposure, unit	Outcome -data source (No. of SNPs)	Method	OR (95% CI)	*P* value	Conditional F-statistic
**Ferritin, SD**	**Liver steatosis**	**Adjusted for FI**			
	- UKB (55)	MVMR IVW	1.57 (1.26, 1.95)	5.85E-05***	22
	- deCODE (49)	MVMR IVW	1.25 (0.73, 2.14)	0.413	22
	- FinnGen (54)	MVMR IVW	1.36 (0.80, 2.31)	0.249	22
	- INTERMOUNTAIN (55)	MVMR IVW	1.01 (0.75, 1.36)	0.93	21
		**Meta-analysis(I^2=13%)**	**1.34 (1.14, 1.57)**	**3.72E-04*** **	

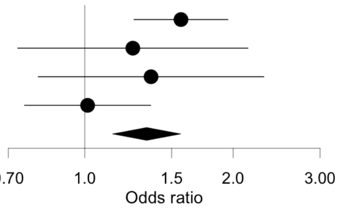

Fig. S6 The associations of fasting insulin, ferritin, and liver iron in liver steatosis with mediators and exposures adjustment using multivariable Mendelian randomization analysis. (A) The associations of fasting insulin in steatosis adjusting for ferritin to assess the direct effect; (B) The associations of ferritin in steatosis adjusting for fasting insulin to correct for horizontal pleiotropy. Steatosis, log odds (*n* = 885,701 including 9,491 cases and 876,210 controls) included UKB (5,921 cases), deCODE (785 cases), FinnGen (651 cases), and INTERMOUNTAIN (2,134 cases). FI, fasting insulin; No. of SNPs, number of single nucleotide polymorphisms; MVMR, multivariable Mendelian Randomization; IVW, Inverse-variance weighting; I^2, degree of heterogeneity; OR, odds ratio; 95% CI, 95% confidence interval. **P* value <0.05, ***P* value <0.01, ****P* value <0.001.
